# Thermally Stable Anthracene-Based 2D/3D Heterostructures for Perovskite Solar Cells

**DOI:** 10.1021/acsami.4c17382

**Published:** 2024-12-27

**Authors:** Kathryn Bairley, Junxiang Zhang, Damara G. Dayton, Courtney Brea, Pattarawadee Therdkatanyuphong, Stephen Barlow, Guoxiang Hu, Michael F. Toney, Seth R. Marder, Carlo A. R. Perini, Juan-Pablo Correa-Baena

**Affiliations:** † School of Materials Science and Engineering, Georgia Institute of Technology, North Ave NW, Atlanta, Georgia 30332, United States; ‡ Renewable and Sustainable Energy Institute (RASEI), University of Colorado Boulder, Boulder, Colorado 80309, United States; § Materials Science and Engineering Program, University of Colorado Boulder, Boulder, Colorado 80309, United States; ∥ Department of Chemistry and Biochemistry, Queens College of the City University of New York, New York, New York 11367, United States; ⊥ Department of Materials Science and Engineering, School of Molecular Science and Engineering, Vidyasirimedhi Institute of Science and Technology, Wangchan, Rayong 21210, Thailand; # Department of Chemical and Biological Engineering, University of Colorado Boulder, Boulder, Colorado 80309, United States; ¶ Department of Chemistry, University of Colorado Boulder, Boulder, Colorado 80309, United States; ∇ School of Chemistry and Biochemistry, Georgia Institute of Technology, North Ave NW, Atlanta, Georgia 30332, United States

**Keywords:** perovskite solar cells, thermal stability, bulky cations, surface treatment, 2D/3D Heterostructures

## Abstract

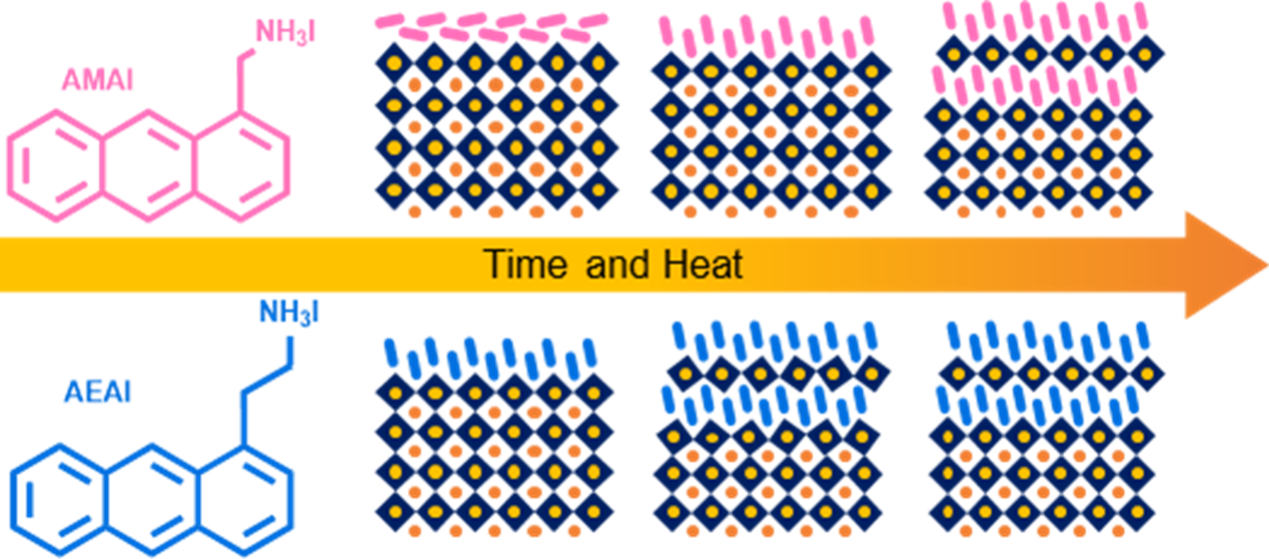

Bulky organic cations are used in perovskite solar cells as a protective barrier against moisture, oxygen, and ion diffusion. However, bulky cations can introduce thermal instabilities by reacting with the near-surface of the 3D perovskite forming low-dimensional phases, including 2D perovskites, and by diffusing away from the surface into the film. This study explores the thermal stability of Cs_0.09_FA_0.91_PbI_3_ 3D perovskite surfaces treated with two anthracene salts—anthracen-1-ylmethylammonium iodide (AMAI) and 2-(anthracen-1-yl)ethylammonium iodide (AEAI)—and compares them with the widely used phenethylammonium iodide (PEAI). The steric hindrance of AMAI limits the interaction of its NH_3_^+^ head with the perovskite lattice, relative to what is seen with AEAI and PEAI. As a result, AMAI requires more thermal energy to convert the 3D perovskite surface to a 2D perovskite. Annealing of perovskite surfaces treated with the iodide salts results in decreased power conversion efficiencies (PCEs) for PEAI and AEAI, while a PCE enhancement is observed for AMAI. Importantly, AMAI-treated devices show enhanced stability upon annealing of the film and a 100% yield of working pixels after a high-temperature stability test at 85 °C, representing the most reliable device configuration among all those studied in this work. These results reveal the potential of AMAI as a scalable surface treatment.

## Introduction

Perovskite solar cells (PSCs) have been demonstrated to achieve a lab-scale device power conversion efficiency (PCE) of over 26%.^1^ While PSCs rival silicon cells in PCE, many challenges must still be addressed to enable their commercialization, including stability, scalability, and reproducibility.^2−4^ Moisture, oxygen, and light exposure can all induce degradation of the perovskite active layer. Current research has been focused on addressing these limitations through interface modifications, studying the interactions that occur between layers.^5−7^ Especially, the addition of halide salts of bulky cations for treating the surface of perovskite (the active layer) has proven effective in enhancing device efficiency and, in certain instances, stability.^8−14^ Bulky cations are defined as those too large to form a 3D perovskite crystal. Upon treating the surface of a 3D perovskite film, bulky cations can replace some of the smaller organic cations in the film, inducing the separation of the octahedra in the 3D lattice and the formation of lower dimensional phases instead such as Ruddlesden–Popper (A′_2_A_*n*–1_B_*n*_X_3*n*+1_) and Dion–Jacobson (A″A_*n*–1_B_*n*_X_3*n*+1_) structures, where A′ and A″ are bulky ammonium or diammonium cations, respectively, A is a cation that fits in the 3D perovskite lattice, B is a divalent metal cation, and X is a halide anion. These low-dimensional structures consist of *n* layers of corner-sharing lead halide octahedra situated between the bulky organic cations and are commonly referred to in the literature as “2D perovskites”. Two-dimensional perovskites can serve as a protective barrier against moisture, oxygen, and ion diffusion as is the case for monoammonium cations having hydrophobic organic “tails”.^8,14^ However, the addition of bulky organic cations does not always induce an immediate surface conversion. Thermal annealing is often necessary for the near-surface to undergo a phase transition via substitution of smaller A cations with A′ bulky cations. The structural changes in thin films treated with bulky ammonium cations can proceed at a temperature as low as 55 °C, leading to diffusion of the bulky cations from the surface into the 3D bulk.^13,15,16^ The changes in thickness, structure, and heterogeneity of the film’s surface at this 2D/3D interface can lead to the formation of an insulating layer and to charge-transport losses.^9,17−20^

Herein, we explore the impact of the strength of π–π stacking between bulky organic cations and the interaction between the organic anchoring group (*i.e*., the ammonium part of the molecule) and the lead halide octahedra on the thermal stability of solar cells incorporating 3D perovskite films treated with ammonium capping layers. We use X-ray photoelectron spectroscopy (XPS) and grazing-incidence wide-angle X-ray scattering (GIWAXS) to understand how the chemical composition and structure of the films and surface layers evolve under thermal annealing. We correlate these findings with the characterization of complete solar cells via current density measurements as a function of applied bias (*J*–*V* scans), maximum power point tracking, and long-term stability measurements. We observe a correlation between the steric hindrance of the cations and slower conversion of the perovskite surface. Among the bulky cations anthracen-1-ylmethylammonium iodide (AMAI), 2-(anthracen-1-yl)ethylammonium iodide (AEAI), and 2-phenylethylammonium iodide (PEAI) used for the surface treatment, AMAI shows the slowest conversion to the 2D phase. AMAI-treated devices exposed to thermal stress also reveal a PCE enhancement and increased reliability during maximum power point tracking (MPPT). Such observations highlight AMAI as a promising scalable and more thermally stable capping layer.

## Results and Discussion

### Experimental Design

**Figure 1:**
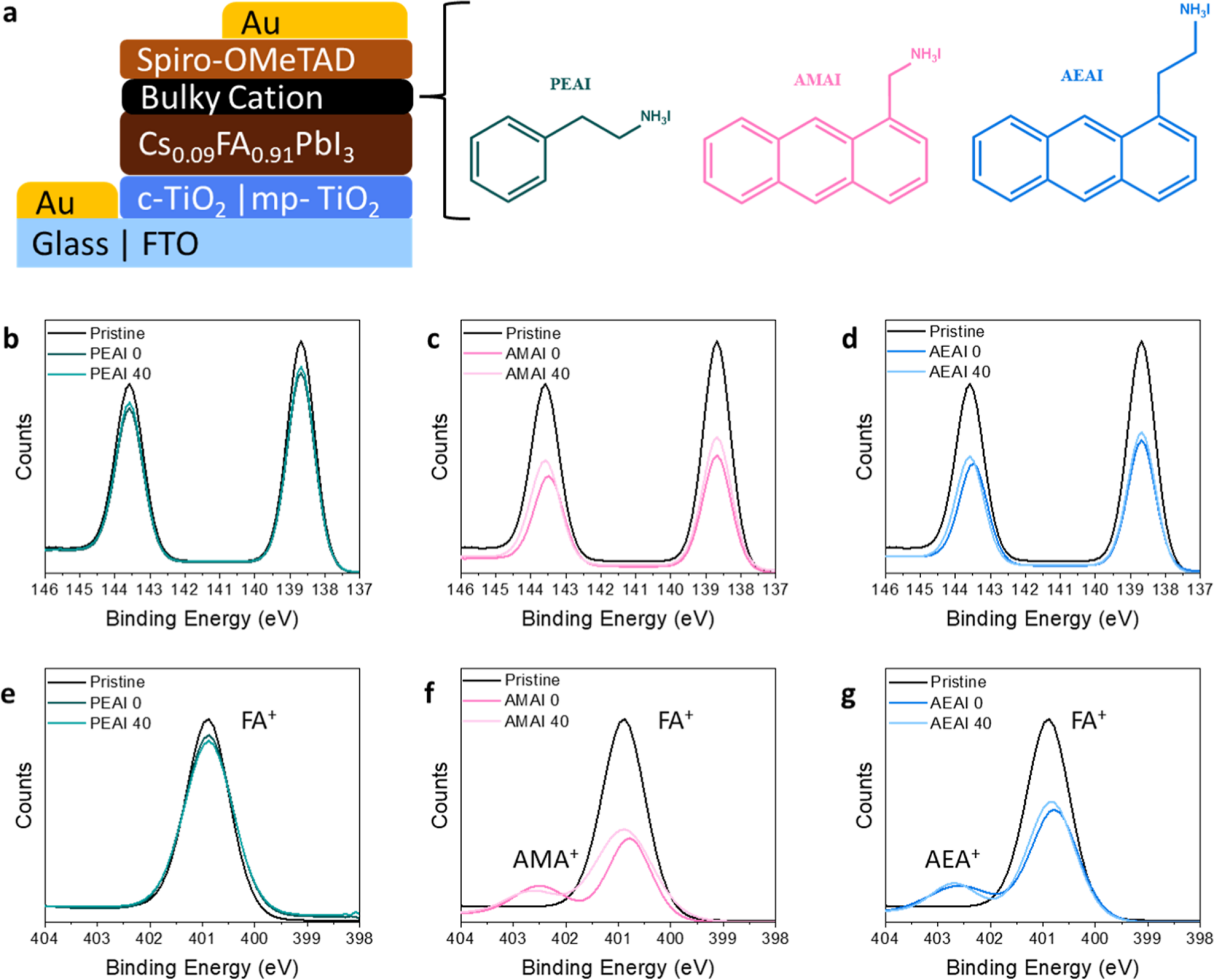
(a) Schematic representation of the n–i–p device architecture and the chemical structures of the bulky ammonium salts. XPS elemental scans of (b–d) Pb 4f and (e–g) N 1s for perovskite films treated with PEAI (b,e), AMAI (c,f), and AEAI (d,g). The pristine Cs_0.09_FA_0.91_PbI_3_ film spectrum is reported in black for all plots. The annealing time in minutes at 100 °C is after the name of the deposited salt (0 or 40 min).

To study the impact of π–π stacking strength and of the interaction between the anchoring group and lead halide octahedra on the thermal stability of perovskite solar cells incorporating a 3D perovskite layer treated with ammonium capping layers, we coat with AMAI, AEAI, and PEAI a 3D Cs_0.09_FA_0.91_PbI_3_ film, where FA^+^ is formamidinium, as shown in Figure 1a. The treated films are studied before and after an annealing treatment at 100 °C for 40 min, to reveal the structural transformations of the surface layer.^15^ Duration of 40 min is chosen instead of the shorter annealing times commonly used in the literature to capture structural changes that could happen with slower dynamics.^15,20,21^

### Film Composition

The surface chemical composition of the pristine Cs_0.09_FA_0.91_PbI_3_ perovskite versus films treated by spin-coating 1 mg/mL solutions of PEAI, AMAI, and AEAI in isopropanol can be determined with the use of X-ray photoelectron spectroscopy (XPS). The synthesis and chemical characterization of the AMAI and AEAI salts used in this work are reported in the Material Synthesis section and Figures S1–S8 in the Supporting Information. XPS provides information about the elemental composition of the near-surface film (10 nm) as well as the chemical bonds present by examining the energy of photoejected near-surface core electrons and their relative abundance. Figure 1b–g depicts the evolution of the Pb 4f and N 1s XPS peaks as the capping layers are deposited and annealed, illustrating the effect of annealing on the surface composition for each of the capping layers. The I 3d, C 1s, and Cs 3d peak signals are reported in Figures S9–S11. Table S1 reports the surface composition of the films as retrieved from the XPS elemental scans.

The Pb 4f doublet in the pristine Cs_0.09_FA_0.91_PbI_3_ perovskite film is seen at binding energies of 143.6 and 138.7 eV, consistent with the binding energies observed for Pb^2+^ in similar perovskite compositions.^15^ The change in binding energy of these peaks is comparable with the measurement error for the films treated with the bulky cations, indicating that the change in the chemical environment is minimal (Figure 1b–d). While we expect the peaks to decrease in intensity upon deposition of the capping layer, the limited change in intensity of the Pb 4f peaks after PEAI treatment suggests the formation of a very thin capping layer, possibly because of the diffusion of PEA^+^ molecules into the bulk of the film, as will be discussed later. For the unannealed AMAI and AEAI films, the Pb 4f signal decreases to half the intensity of those seen for the pristine film (Figure 1c,d). The greater decrease in peak intensity for the bulkier anthracene cations suggests the formation of thicker layers of organic salts at the top 3D perovskite film surface than in the case where PEAI is used. When the films treated with AMAI and AEAI are annealed at 100 °C, the Pb 4f peaks slightly increase in intensity with respect to the corresponding nonannealed films, consistent with the potential reaction of anthracene cations at the surface with the 3D perovskite to form 2D perovskite phases (vide infra).

The N 1s peak has a binding energy of 400.9 eV in the pristine Cs_0.09_FA_0.91_PbI_3_ perovskite film that can be attributed to N of FA^+^.^22^ The peak position is unchanged in the PEAI-treated films, with a slight decrease in intensity (Figure 1e), following the same trend as the Pb 4f spectrum. An ammonium (R-NH_3_^+^) species such as that in the bulky cation would be expected to show a N 1s peak at higher BE since that N bears a full positive charge, whereas those of FA^+^ bear approximately half a positive charge each; however, no such higher BE peak is seen in the case of PEAI deposition, indicating that the amount of PEA^+^ cations remaining at the surface is below the detection limit of our measurement.^22,23^ Such low concentration could be rationalized assuming the deposited PEAI molecules diffused toward the bulk of the film, as will be discussed later. In the AMAI and AEAI films, there is a reduction in the intensity of the FA^+^ peak and the formation of a prominent second R-NH_3_^+^ peak. Since XPS is a surface-sensitive technique, the decrease in intensity of the FA^+^ peak corroborates the hypothesis that these molecules formed thicker layers on the surface of the 3D film with respect to that when PEAI is used.

As the films treated with AMAI and AEAI are annealed, the FA^+^ peak intensity increases, while the R-NH_3_^+^ peak decreases. In the PEAI-treated films, the intensity of the FA^+^ peak is relatively unchanged following thermal treatment. Based on the small changes observed in the N 1s XPS spectrum, it is hypothesized that the majority of AMA^+^ and AEA^+^ cations remain at the surface of the film during thermal annealing, while some FA^+^ moves to the surface. Overall, XPS of the films suggests that AMAI and AEAI molecules largely remain at the surface of the perovskite film upon annealing and indicates that the amounts of PEAI at the surface are below the detection limit of the technique even before the annealing treatment.

### Polycrystalline Thin-Film Structure

**Figure 2:**
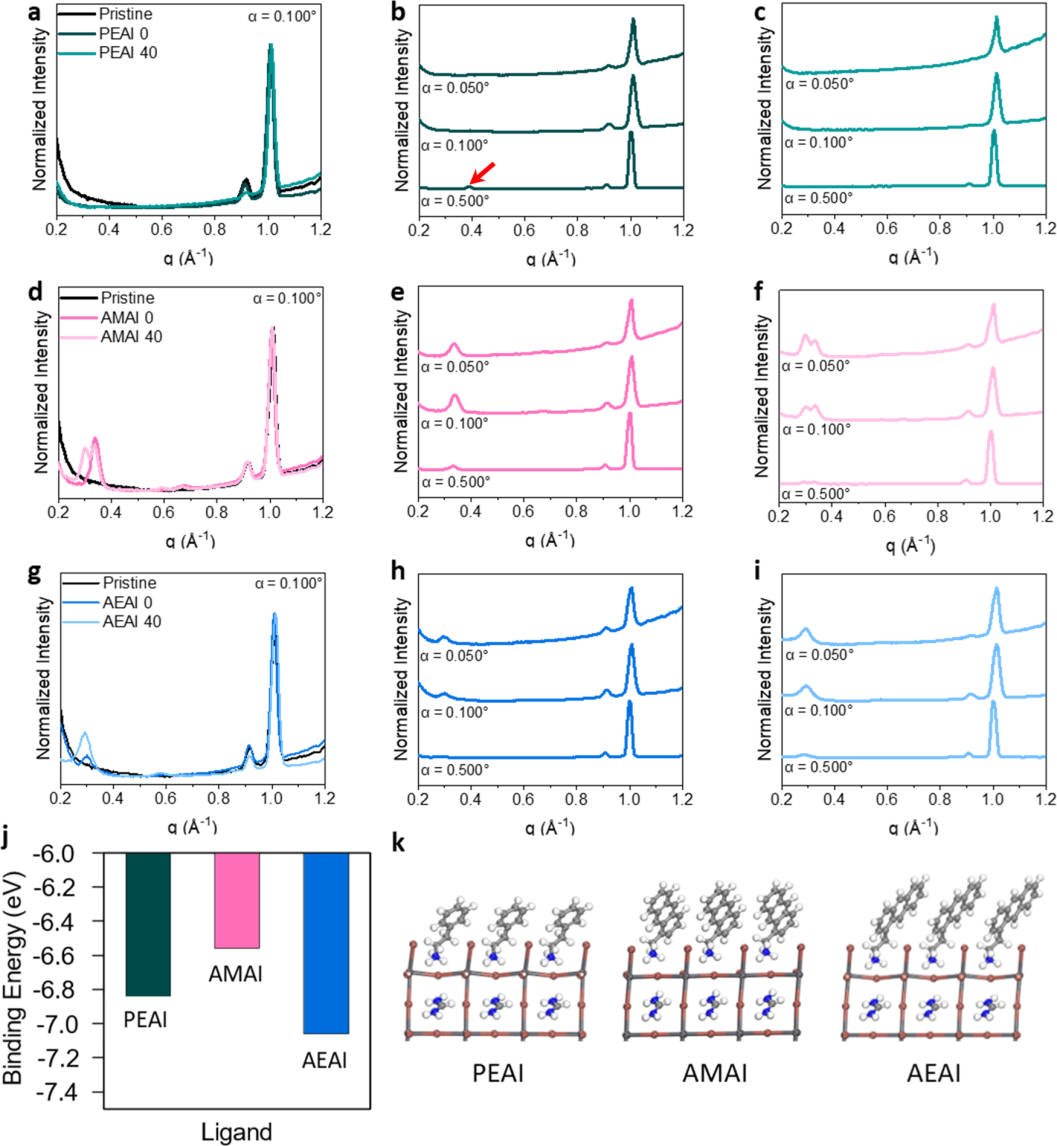
Radial integration of 1D scattering profiles for −20° < χ < 20° at an incidence angle of α = 0.100° for (a) PEAI, (d) AMAI, and (g) AEAI films with and without annealing in comparison to the pristine perovskite film. Radial integration of 1D scattering profiles for −20° < χ < 20° as a function of incident angle for (b) PEAI 0, (c) PEAI 40, (e) AMAI 0, (f) AMAI 40, (h) AEAI 0, and (i) AEAI 40. All data are normalized with respect to the 3D perovskite peak maximum intensity at *q* = 1.0 Å^–1^ and are offset for clarity. A red arrow indicates the diffraction peak from PEA_2_PbI_4_. (j) Binding energy of the three cations from DFT calculations. (k) Optimized atomic structures for monolayers of bulky cations on top of a 3D FAPbI_3_ perovskite lattice.

Grazing-incidence wide-angle X-ray scattering (GIWAXS) was performed to examine the crystal structure of the near-surface and bulk of the films and annealing-induced phase transformations.^24^ The full GIWAXS patterns of the samples are reported in Figures S12 and S13, for the reference and treated films, respectively. As the strong diffraction peaks from the low-dimensional phases in Figure S13 are localized around the out-of-plane direction *q*_*z*_, we present the 1D scattering profiles along *q*_*z*_ in Figure 2. We retrieved these profiles by radially integrating the out-of-plane signal between the azimuthal angles −20° < χ < 20°, for three incidence angles: α = 0.050°, 0.100°, and 0.500°. As the incidence angle increases, the X-rays probe deeper into the bulk of the film, with the estimated penetration depths <3 nm at 0.050°, 3–4 nm at 0.100°, and 225 nm at 0.500°.^13^ The 1D radial profiles retrieved from integrating the signal for −90° < χ < 90° are presented in Figures S12 and S14 and follow similar trends in intensity as those in Figure 2. In Figure 2, the peaks at 1.00 and 0.91 Å^–1^, visible in all the patterns, are attributed to the cubic phase of Cs_0.09_FA_0.91_PbI_3_ and PbI_2_, respectively, while the peaks at lower *q* values can be attributed to the low-dimensional structures formed by the reaction of the 3D film with the bulky organic salts.^24−28^

There are minimal differences between the PEAI-treated and pristine films at α = 0.050° and α = 0.100°, with no peaks visible at the expected *q* values for the *n* = 1 and *n* = 2 Ruddlesden–Popper phases (Figures 2b,c, S12b). For the unannealed PEAI film at α = 0.500° (Figure 2b), a very weak peak is present at 0.39 Å^–1^ and is attributed to the diffraction of the 002 plane of the PEA_2_PbI_4_*n* = 1 Ruddlesden–Popper 2D perovskite (Figure S15). The presence of a 2D perovskite peak deeper in the film may suggest that PEA^+^ diffuses into the bulk before incorporating into the perovskite lattice, which is in agreement with the XPS results of PEAI-treated films and with previous studies that showed diffusion of PEA^+^ cations along grain boundaries and conversion of the grain surfaces to *n* = 1 Ruddlesden–Popper structures.^15,29^ Upon annealing of the film, the 2D perovskite peak at α = 0.500° disappears, which shows a loss of any crystalline PEA_2_PbI_4_ in the probed volume.

Films treated with AMAI show a prominent peak at 0.34 Å^–1^, while a peak at 0.30 Å^–1^ is visible in AEAI-treated films. In both cases, those peaks are attributable to lower dimensional phases (Figure 2d,g). A second peak at 0.30 Å^–1^ is also present in the annealed AMAI films in Figure 2d, f, and this feature was also observed in the powder XRD patterns of pristine 2D films deposited on glass by mixing PbI_2_ and AMAI in a 2:1 ratio (Figure S15). As the presence of the two peaks is independent from interaction with Cs^+^ and FA^+^ in the 3D film, and as the two peaks vary in intensity independently, we attribute the presence of the two peaks in AMAI-treated films to the formation of two different low-dimensional polymorphs upon reaction with the underneath perovskite layer, possibly due to different tilts in the anthracene moieties.^30^ Since it is expected that the AMAI and AEAI 2D phases have similar interplanar spacing with a difference of only ∼0.5 Å and that the peak at 0.30 Å^–1^ is dominant in the powder XRD pattern of the pristine AMAI 2D film (Figure S15), we hypothesize that the lower *q*-value peak at 0.30 Å^–1^ is the diffraction signal for a more thermodynamically stable 2D perovskite phase, which we will call “phase 2” and that the larger *q*-value peak is evidence of an intermediate phase that occurs during film formation, which we will call “phase 1”. In other words, heating is required to form the more stable 2D phase, suggesting the presence of an energetic barrier for stable 2D phase formation. However, it is important to note that the two phases coexist after thermal treatment, indicating incomplete conversion.

When probing the bulk of the AMAI- and AEAI-treated films by using an incidence angle α = 0.500°, the relative intensity of the 2D perovskite peak to the 3D perovskite peak decreases with respect to the surface-sensitive measurements (α = 0.050°, α = 0.100°) (Figures 2e,h, S16). This observation reveals that AMA^+^ and AEA^+^ form crystalline 2D phases near the surface, differently than in the case of PEA^+^, where diffraction from the 2D phase is observed only when probing the bulk. Annealing of the films results in increased signal from low-dimensional phases but does not change the intensity trends as a function of the incidence angle (Figure S16). In the annealed AMAI-treated film, the ratio of the phase 1 and 2 peaks evolves as the incidence angle increases (Figure 2f). At α = 0.050°, the phase 2 peaks have stronger diffraction than that of phase 1 and reduce in relative intensity at greater incidence angles until the signals are almost equal at α = 0.500°. This trend could be explained if we assume that the conversion from 3D to 2D as initiated by AMAI undergoes a transition from phase 1 to phase 2 during film formation at elevated temperatures and assuming that AMAI diffuses into the bulk with annealing. As AMAI must first diffuse into the bulk with annealing, the conversion to the 2D phase in the bulk will be slower with respect to what was observed at the surface, leading to a change in the relative intensities of the two peaks.

**Figure 3:**
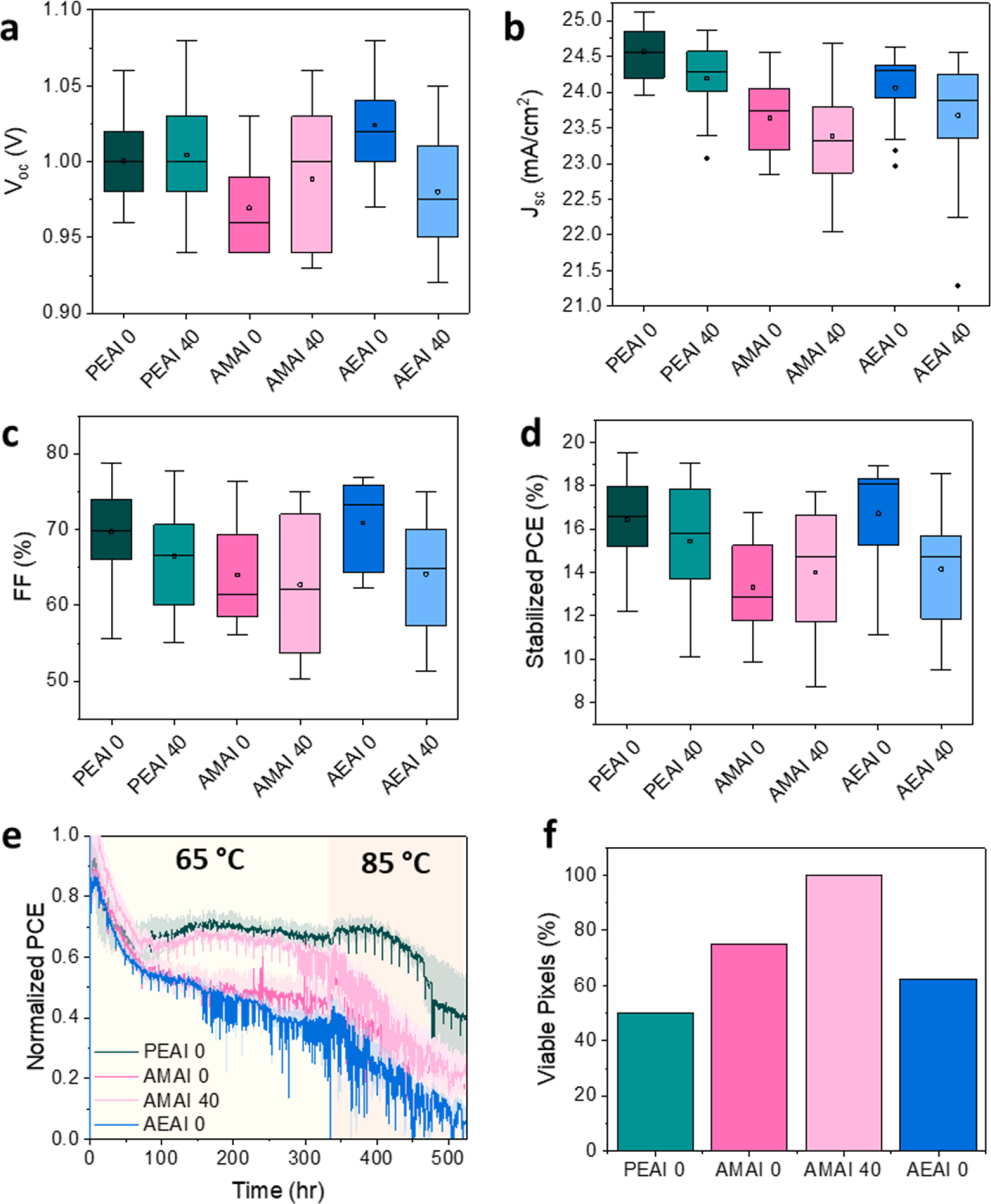
Box plots of (a) open-circuit voltage, (b) short-circuit current density, (c) fill factor, and (d) stabilized power conversion efficiency for each capping variation. Values for plots (a–c) are based on the reverse *J*–*V* scans. (e) Thermal stability of devices with varied capping layers and postdeposition thermal treatments. (f) Percentage of pixels in each cell that maintained nonzero PCE throughout the stability measurements.

In addition to the trends presented in Figure 2, sample-to-sample variation of the 1D scattering patterns was observed, particularly in the AMAI-treated samples (Figure S17e–h). We attribute these variations to nonuniformity of each film rather than across the films due to the wide distribution in device performance (Figures 3, S18). The PEAI- and AEAI-treated films also exhibit sample-to-sample variation (Figure S17a–d,i–l), but to a lesser extent, which is complemented by narrower distributions in device performance (Figures 3, S18).

Finally, the crystallite size of the AMAI and AEAI 2D capping layers was estimated from the peak width of the radial integration of 1D scattering profiles for −20° < χ < 20° at an incident angle of α = 0.100° (Table S2). The calculation of the domain size using the Scherrer equation shows that the AEAI capping layer decreases in crystallite size (a proxy for film thickness) with the addition of the heat treatment. The variation in crystallite size for the AMAI layer is more difficult to interpret due to the appearance of a second peak from low-dimensional phases. Vertical phase segregation for the two phases would indicate an increased crystallite size of the 2D layer at the surface, while lateral phase segregation would mean that the crystallite size is almost unchanged after annealing. Vertical phase segregation would be consistent with both the ratio of 2D to 3D phases present in each film (Figure S16), the small variations in Pb 4f and N 1s signals in XPS, and the resulting device performance (Figures 3, S18 and 19), with a thicker capping layer improving efficiency and stability.

Density functional theory (DFT) calculations were performed to further elucidate how the interactions of the bulky cations with the surface and with one another could impact the formation of 2D phases and the diffusion of the bulky cations into the bulk. Specifically, we calculated the binding energy for the cations on the surface of 3D perovskite at one monolayer coverage (Figure 2j). This captures both the interaction between neighboring ligands and the interaction of NH_3_^+^ groups with A-site cation vacancies at the surface. As the interaction of the NH_3_^+^ group with the Pb–I lattice is the same for AEAI and PEAI, we ascribe the increased surface binding energy of AEAI to a stabilization through π–π interactions with neighboring molecules also bound to the surface. Conversely, the less negative binding energy of AMAI compared to AEAI can be ascribed to a reduced penetration of the NH_3_^+^ group into the lattice, which is limited by the steric hindrance of the anthracene group (Figure 2k). We note that for all the cations, the binding energies are more negative than the one of FA^+^ on the surface (−5.89 eV). Therefore, it is favorable for all cations to substitute FA^+^ at the surface of the 3D film. Reduced penetration of the NH_3_^+^ group into the Pb–I lattice has been linked with an increased barrier to converting the 3D lattice into a 2D structure, while a more negative binding energy has been correlated with greater passivation effects, resulting in improved PCE and *V*_OC_.^23^ Based on these considerations and on previous literature reports, we expect AMAI to show the slowest near-surface phase transition dynamics and AEAI to provide the most effective passivation among the three salts studied.^23^

### Device Performance and Thermal Stability

We then proceeded to correlate the information collected near the salt-treated interfaces with the performances of complete solar cells. We fabricated n–i–p architectures constituting glass | fluorine-doped tin-oxide (FTO) | compact-TiO_2_ | mesoporous-TiO_2_ | Cs_0.09_FA_0.91_PbI_3_ | (bulky cation)-I | 2,2′,7,7′-tetrakis(di(4-methoxyphenyl)amino)-9,9′-spirobifluorene (Spiro-OMeTAD) | Au (Figure 1a). We then measured open-circuit voltage (*V*_OC_), short-circuit current density (*J*_SC_), fill factor (FF), power conversion efficiency (PCE), stabilized PCE (PCE after maximum power point tracking for 1 min, Figure S18), and hysteresis, extracted from the forward and reverse *J*–*V* scans of solar cells made for each variation. Performing these calculations on 24 pixels for each of the six device variations allows for statistical comparisons to be made between the bulky cations and the effect of annealing (Figures 3a–d, S19) and to compare the trends among the maximum, median, and median of absolute deviation values (Table S2). We report *V*_OC_, *J*_SC_, FF, and stabilized PCE in Figure 3, while the PCE and hysteresis are reported in Figure S19 for completeness.

The *V*_OC_ trends for the three cations before annealing follow the same trend as the bulky cation binding energies and suggest greater passivation effects (i.e., reduced nonradiative recombination) for cations with stronger binding energies (Figures 3a,b, and 2j), in agreement with previous literature.^23^ The median open-circuit voltage for PEAI-treated devices is 1.00 V, against 0.96 V for AMAI and 1.02 V for AEAI. Analogously, the stabilized PCEs are 16.3% for PEAI, 11.8% for AMAI, and 15.8% for AEAI, while the median FF values are 69.8%, 72.5%, and 61.2% for PEAI, AEAI, and AMAI, respectively. The *J*_SC_ values of PEAI (24.6 mA/cm^2^) and AEAI (24.3 mA/cm^2^) are comparable, decreasing to 23.7 mA/cm^2^ for AMAI. The *J*_SC_ value decreases for all devices upon annealing. It is worth noting that the distribution of device performances broadens for AMAI-treated devices, which could stem from the presence of two different low-dimensional phases at the interface with the 3D film.^31^ We do not expect any of these performance changes to be due to variations in the surface microstructure of the films, given the low concentration of cations used in this work.^15^

Upon annealing for 40 min at 100 °C, the median-stabilized PCE of devices incorporating PEAI and AEAI decreases to 15.8% and 14.3%, respectively, while the median-stabilized PCE of devices incorporating AMAI increases to 14.7%. These variations are mainly ascribed to losses in *V*_OC_ and FF for the PEAI and AEAI devices, while *V*_OC_ is enhanced for the AMAI devices and FF remains unchanged. These differences can again be correlated to the interaction of the cations with the lattice, as suggested by DFT. Because of reduced interaction with the surface and strong π–π interactions, AMAI might be likely to aggregate instead of reacting with the 3D perovskite surface. During thermal annealing, the ions have more energy to overcome the π–π interactions between the anthracene moieties, allowing the diffusion of AMA^+^ and I^–^ on the surface via the formation of a 2D layer, reducing nonradiative carrier recombination in the device. Instead, the stronger interaction with the lattice of PEAI and AEAI leads to the conversion of the perovskite near-surface region to low-dimensional phases upon deposition. With annealing, PEAI shows loss of crystallinity in the top 225 nm probed by GIWAXS, while AEAI converts more of the 3D metal halide perovskite to low-dimensional phases. We speculate that the strong interaction of AEAI with the surface and the resulting breaking of bonds could be related to the lower performances produced after annealing.

To analyze the effects of the anthracene salts and annealing on hysteresis, we present box plot summaries of open-circuit voltage, short-circuit current density, fill factor, and power conversion efficiency during forward and reverse scans, as well as forward and reverse *J*–*V* scans (Figures S20–S21). Samples treated with both PEAI and AMAI exhibit an overall decrease in hysteresis upon annealing, suggesting thermally assisted defect passivation and favorable changes in the band structure at the interface. Meanwhile, samples treated with AEAI reveal increased hysteresis after thermal treatment. The different evolution in hysteresis before and after annealing for AEAI and AMAI might be related to the different structures formed at the surface by the two bulky cations. Overall, the devices with PEAI capping layers offer the best initial PCE values but degrade with annealing at 100 °C for 40 min and have lower median-stabilized PCE than AEAI. AEAI-treated devices improve the median-stabilized PCE and *V*_OC_ of devices, in agreement with the trends seen in binding energies, but experience a greater decrease in performance following thermal treatment. Devices treated with AMAI have the lowest initial performance values but improve in PCE upon annealing. The added thermal energy is hypothesized to break apart the AMAI aggregates and to promote conversion to a crystalline 2D perovskite.

Prompted by the different behavior of the salt-treated films under high temperature, we proceeded to investigate device performance under thermal stress during photovoltaic operation. An AMAI film annealed at 100 °C for 40 min was included in the experiment given that thermal energy is required for this cation to promote the reaction with surface sites. We heated unencapsulated solar cells to 65 °C in a N_2_ environment and monitored the evolution of their performances as a function of time. The normalized efficiency as a function of time is plotted in Figure 3e. At 65 °C, devices treated with PEAI, AMAI, and AEAI follow similar degradation rates for the first 24 h. After the first day, the PEAI-treated device stabilizes to 70% of initial PCE while the unannealed AMAI- and AEAI-treated devices continue to degrade, reaching 50% and 40% of initial PCE after 335 h at 65 °C, respectively. Increasing the temperature to 85 °C further reduces the efficiency of all film variations, with PEAI having the slowest degradation. These trends are different than that observed when films are annealed before spiro-OMeTAD and Au deposition and suggest other degradation mechanisms are at play when complete devices are exposed to high temperature (e.g., iodine diffusion and reaction with the Au electrode). Interestingly, the postannealed AMAI-treated device (AMAI 40) maintained 65% of initial PCE after 335 h at 65 °C, comparable to the stability exhibited by the device made with a PEAI capping layer. We attribute this improvement in stability to enhanced surface passivation if a 2D perovskite phase is formed before a complete device is exposed to thermal stress.

We complete our analysis of thermal stability by comparing the yield of pixels that survived the thermal stress (Figures 3f, S22). Here, we refer to this yield as the percentage of “viable pixels” with respect to the pixel total. Viable pixels are those that maintained nonzero PCE throughout the stability measurements, while pixels not working initially or those that stopped working during testing are excluded (Figure S22). In the devices treated with PEAI, a large fraction of pixels stopped working before the end of the stability measurement (loss of contact or 0% PCE). For devices treated with AMAI and AEAI, a greater proportion of pixels were deemed “viable” for analysis than those treated with PEAI (Figure 3f). Only half of the pixels were considered viable in the PEAI-treated device, while all pixels consistently worked in the annealed AMAI-treated device. As such, AMAI shows promise for near-surface passivation, to enable scalable and reproducible devices while granting similar thermal stability as the best pixels made with the state-of-the-art capping layer, PEAI.

## Conclusions

This work compared the performance and stability of hybrid organic–inorganic metal halide perovskite solar cells incorporating capping layers derived from the treatment of Cs_0.09_FA_0.91_PbI_3_ with PEAI, AMAI, and AEAI. The bulkier anthracene-derived cations showed slower diffusion and conversion to two dimensions of the 3D perovskite surface than PEAI. The anthracene-based salt AEAI enabled the best median PCEs when incorporated in a solar cell structure, before annealing, in agreement with the binding energy trends predicted by DFT. Upon annealing at 100 °C, device efficiency is improved for AMAI-treated devices, which we ascribe to the formation of a 2D perovskite phase and to the diffusion of the cation into the bulk of the absorbing layer. The annealed AMAI-treated solar cells revealed the best yield of pixels surviving a thermal stability measurement at 65 °C and 85 °C, among all bulky cations compared in this study, and PCE decay comparable to the best pixels of PEAI, proving AMAI as a promising material for interface passivation and improving the stability and scalability of the solar cells.

## Experimental Section

### Material Synthesis

The synthesis of the anthracene ammonium iodide derivatives was carried out in a similar, standard procedure, illustrated in Figure S1.

#### 1-Chloroanthracene (**1**)

1-Chloroanthracene (**1**) was synthesized by following the literature procedure,^32^ obtaining a pale-yellow solid (50% yield). ^1^H NMR (400 MHz, CDCl_3_): δ 8.88 (s, 1H), 8.47 (s, 1H), 8.16–8.07 (m, 1H), 8.07–8.01 (m, 1H), 7.95 (d, *J* = 8.5 Hz, 1H), 7.60 (dd, *J* = 7.3, 1.1 Hz, 1H), 7.58–7.52 (m, 2H), 7.38 (dd, *J* = 8.6, 7.1 Hz, 1H). HRMS-ESI, calcd. for C_14_H_9_Cl^+^ [M^+^]^+^ 212.0387; found, 212.0399.

#### Anthracene-1-carbonitrile (**2**)

1-Chloroanthracene (0.047 mmol), Pd(OAc)_2_ (0.01 mmol), RuPhos (0.019 mmol), and K_4_[Fe(CN)_6_·3H_2_O] (0.235 mmol) were added into a two-neck round-bottom flask. The mixture was evacuated and then refilled with nitrogen for three cycles. A 3:1 ratio of toluene:H_2_O was added to the dry mixture. The solution was stirred and heated at 70 °C for 24 h. After the reaction cooled to room temperature, the reaction was extracted with dichloromethane (DCM) and water; the organic layers were combined, concentrated in vacuo, and purified with column chromatography on silica gel using CH_2_Cl_2_/hexane (1/1, v/v) as the eluent to obtain a yellow solid product (95% yield). ^1^H NMR (400 MHz, CDCl_3_): δ 8.83 (s, 1H), 8.54 (s, 1H), 8.26 (d, *J* = 8.6 Hz, 1H), 8.18–8.10 (m, 1H), 8.10–8.03 (m, 1H), 8.01–7.94 (m, 1H), 7.66–7.55 (m, 2H), 7.51 (dd, *J* = 8.6, 6.9 Hz, 1H). HRMS-ESI, calcd. for C_15_H_10_N^+^ (MH^+^) 204.0808; found, 204.0818.

#### Anthracene-1-ylmethylammonium Chloride (**3**)

To a solution of anthracene-1-carbonitrile (3.941 mmol) in THF (16 mL), BH_3_·THF (11.82 mmol) was slowly added over 10 min under nitrogen atmosphere. The reaction was heated to reflux for 3 h. After the reaction cooled in an ice bath, distilled water (12 mL) was added cautiously, followed by the addition of concentrated aqueous HCl (12 mL) over 5 min. Then, the reaction was brought to reflux again for 24 h. After the reaction cooled to room temperature, THF was evaporated out by a rotary evaporator without heat. The solid residue was washed with water and then diethyl ether to obtain a yellow solid product (66% yield). ^1^H NMR (400 MHz, DMSO-*d*_6_): δ 8.84 (s, 1H), 8.68 (s, 1H), 8.54 (br s, 3H), 8.19–8.11 (m, 3H), 7.66–7.54 (m, 4H), 4.67 (s, 2H). ^13^C{^1^H} NMR (101 MHz, DMSO-*d*_6_): δ 131.89, 131.71, 131.56, 130.52, 129.84, 129.19, 128.95, 128.27, 127.44, 127.18, 126.53, 126.44, 125.16, 122.87, 61.80, 40.06. HRMS-ESI, calcd. for C_15_H_11_^+^ (M – NH_3_Cl)^+^ 191.0855; found, 191.0913.

#### *tert*-Butyl (Anthracen-1-ylmethyl)carbamate (**4**)

Anthracene-1-ylmethylammonium chloride (2.459 mmol) was mixed with Boc_2_O (3.688 mmol) in a two-neck round-bottom flask. The mixture was evaluated and then refilled with nitrogen for three cycles. CH_2_Cl_2_ (20 mL) was added to the mixture and then cooled to 0 °C. Triethylamine (4.918 mmol) was slowly added. The reaction was stirred and warmed to room temperature for 2 h. Then, the reaction was extracted with CH_2_Cl_2_ and water, and the organic layers were combined, concentrated in vacuo, and purified with column chromatography on silica gel using 2% EtOAc in CH_2_Cl_2_ as the eluent to obtain a pale-yellow solid product (67% yield). ^1^H NMR (400 MHz, CDCl_3_): δ 8.64 (s, 1H), 8.48 (s, 1H), 8.05 (s, 2H), 7.98 (d, *J* = 7.1 Hz, 1H), 7.54–7.49 (m, 2H), 7.44 (s, 2H), 4.94 (s, 3H), 1.52 (s, 9H). ^13^C{^1^H} NMR (101 MHz, CDCl_3_): δ 155.81, 134.18, 132.02, 131.86, 131.51, 129.61, 128.76, 128.63, 127.91, 127.12, 125.69, 125.67, 125.37, 124.74, 122.39, 79.64, 43.22, 28.46. HRMS-ESI, calcd. for C_20_H_21_NO_2_^+^ (M^+^)^+^ 307.1567; found, 307.1570. Anal. Calcd for C_20_H_21_NO_2_ (%): C, 78.15; H, 6.89; N, 4.56. Found (%): C, 78.16; H, 6.89; N, 4.63.

#### Anthracene-1-ylmethylammonium Iodide (AMAI)

HI (8.135 mmol) was added to a solution of *tert*-butyl (anthracen-1-ylmethyl)carbamate (0.814 mmol) in CH_2_Cl_2_ (8 mL) and MeCN (4 mL). The mixture was heated to reflux for 24 h. After the reaction cooled down to room temperature, diethyl ether was added and stirred for 30 min. The mixture was filtered off to obtain a yellow solid product (89% yield). ^1^H NMR (400 MHz, DMSO-*d*_6_): δ 8.79 (s, 1H), 8.65 (s, 1H), 8.31 (br s, 3H), 8.17–8.06 (m, 3H), 7.62–7.50 (m, 4H), 4.67 (s, 2H). ^13^C{^1^H} NMR (101 MHz, DMSO-*d*_6_): δ 131.83, 131.68, 131.55, 130.36, 129.98, 129.12, 128.82, 128.30, 127.52, 127.21, 126.57, 126.55, 125.13, 122.76, 40.23. HRMS-ESI, calcd. for C_15_H_11_ (M – NH_3_I)^+^ 191.0855; found, 191.0914. Anal. Calcd for C_15_H_14_NI (%): C, 53.75; H, 4.21; N, 4.18. Found (%): C, 54.01; H, 4.36; N, 4.28.

#### *tert*-Butyl (2-(anthracen-1-yl)ethyl)carbamate (**5**)

1-Chloroanthracene (4.704 mmol), potassium β-aminoethyltrifluoroborate (4.704 mmol), Cs_2_CO_3_ (14.111 mmol), Pd(OAc)_2_ (0.235 mmol), and RuPhos (0.470 mmol) were added into a two-neck round-bottom flask. The mixture was evacuated and then refilled with nitrogen for three cycles. The toluene (75 mL) and H_2_O (25 mL) solution was added to the dry mixture. The solution was stirred and heated to reflux for 24 h. After cooling to room temperature, the reaction mixture was filtered through Celite and washed with CH_2_Cl_2_. The organic layers were combined, concentrated in vacuo, and purified with column chromatography on silica gel using 2% EtOAc in CH_2_Cl_2_ as the eluent to obtain a white solid (89% yield). ^1^H NMR (400 MHz, CDCl_3_): δ 8.65 (s, 1H), 8.47 (s, 1H), 8.08 (dt, *J* = 6.6, 3.6 Hz, 1H), 8.05–8.00 (m, 1H), 7.94 (d, *J* = 8.5 Hz, 1H), 7.54–7.48 (m, 2H), 7.42 (dd, *J* = 8.5, 6.7 Hz, 1H), 7.35 (dd, *J* = 6.7, 1.2 Hz, 1H), 4.65 (s, 1H), 3.64 (t, *J* = 6.9 Hz, 2H), 3.44 (t, *J* = 7.0 Hz, 2H), 1.47 (s, 9H). ^13^C {^1^H} NMR (101 MHz, CDCl_3_): δ 155.97, 135.06, 132.17, 131.70, 131.41, 130.46, 128.62, 127.87, 127.59, 127.14, 125.87, 125.58, 125.48, 124.98, 122.39, 79.28, 77.25, 41.02, 33.63, 28.46. HRMS-ESI, calcd. for C_21_H_23_NO_2_^+^ (M^+^)^+^ 321.1703; found, 321.1735. Anal. Calcd for C_21_H_23_NO_2_ (%): C, 78.47; H, 7.21; N, 4.36. Found (%): C, 78.68; H, 7.26; N, 4.46.

#### 2-(Anthracene-1yl)ethylammonium Iodide (AEAI)

AEAI was synthesized by following the procedure described for AMAI. An off-white solid product was obtained (78% yield). ^1^H NMR (400 MHz, DMSO-*d*_6_): δ 8.74 (d, *J* = 1.1 Hz, 1H), 8.59 (s, 1H), 8.14–8.04 (m, 2H), 8.02–7.98 (m, 1H), 7.86 (br s, 3H), 7.56–7.49 (m, 2H), 7.47–7.38 (m, 2H), 3.45 (dd, *J* = 9.1, 6.6 Hz, 2H), 3.22 (dd, *J* = 9.0, 6.7 Hz, 2H). ^13^C{^1^H} NMR (101 MHz, DMSO-*d*_6_): δ 133.65, 132.10, 132.09, 131.79, 131.39, 130.12, 128.75, 128.31, 128.26, 127.48, 126.77, 126.30, 126.27, 125.61, 122.50, 39.78, 30.96. HRMS-ESI, calcd. for C_16_H_16_N^+^ (M – I^+^)^+^ 222.1277; found, 222.1300. Anal. Calcd for C_16_H_16_NI (%): C, 55.03; H, 4.62; N, 4.01. Found (%):C, 54.77; H, 4.71; N, 4.03.

### Device Fabrication

The perovskite solar cells were fabricated with an n–i–p device architecture (Figure 1a). The substrates are made of glass with a patterned fluorine-doped tin oxide (FTO) layer that serves as the bottom transparent electrode. The electron transport layer is composed of compact- and mesoporous-TiO_2_, while Cs_0.09_FA_0.91_PbI_3_ perovskite serves as the active layer. The capping layer consists of one of three ammonium salts: phenethylammonium iodide (PEAI), anthracen-1-ylmethylammonium iodide (AMAI), or 2-(anthracen-1-yl)ethylammonium iodide (AEAI). Doped spiro-OMeTAD was deposited as the hole-transport layer before the gold electrode. The active area of the devices is located toward the center of the sample, while the electrical contacts are on the edges of the device.

The 1 in. × 1 in. patterned FTO glass substrates were cleaned with 10% (v/v) Mucasol (diluted in DI water, Sigma) and scrubbed with a toothbrush for 10 s. After rinsing with DI water, the substrates were sonicated in 2% (v/v) Mucasol (diluted in DI water, Sigma) for 15 min. The substrates were again rinsed with DI water and subsequently sonicated in DI water, acetone (99.5%, Sigma), and IPA (99.5%, Sigma) for 10 min each before being dried with nitrogen gas, one substrate at a time. Immediately before depositing the compact-TiO_2_ layer, the substrates were treated with UV-ozone for 15 min.

The FTO glass substrates were then arranged in two rows on a hot plate under a fume hood. Supporting FTO pieces were placed around the substrates, and glass is used to create a mask over the edges of the substrates before turning on the hot plate. Masking of the edges is required to enable electrical connection to the bottom FTO after the device is completed. After the hot plate reached 450 °C, the substrates were left for 30 min at temperature before depositing the compact-TiO_2_ layer. Meanwhile, 0.480 mL of acetylacetone (99%, Sigma), 0.720 mL of titanium diisopropoxide bis(acetylacetonate) (75% in 2-propanol, Sigma), and 10.8 mL of ethanol (anhydrous, Sigma) were combined in solution and added to a metal spray gun. Spraying with an oxygen flow rate of 3 L/min from a distance of approximately 25 cm, the solution was deposited over the entire length of the hot plate. There was 30 s of wait time between each cycle, with one cycle consisting of running across the hot plate and back to the starting position over the course of about 15 s. Once the solution was depleted, the substrates were left at 450 °C for 30 min before turning the hot plate off and letting it cool to room temperature.

One day before depositing the mesoporous-TiO_2_ layer, a stock solution of 150 mg/mL titanium dioxide paste (19 wt %, Sigma) in ethanol (anhydrous, Sigma) was prepared and stirred with a rod mixer overnight. Prior to spin coating, the edges of each substrate were covered with a clear tape to create a 3 mm mask to allow for deposition in the same area as in c-TiO_2_ spray pyrolysis. A volume of 60 μL of the mp-TiO_2_ solution was then statically deposited before spin coating at 4000 rpm for 10 s. The tape was subsequently removed, and the samples were annealed for at least 10 min at 100 °C. After annealing, the samples were sintered in an enclosed hot plate, slowly heating to 450 °C and holding at that temperature for 30 min. Once the sintering was complete, the samples were left on the hot plate at 150 °C until being brought into the glovebox for perovskite deposition.

The Cs_0.09_FA_0.91_PbI_3_ perovskite solution with 5% excess lead iodide was prepared by mixing 1.05:0.91:0.09 molar ratios of lead iodide (PbI_2_), formamidinium iodide (FAI), and cesium iodide (CsI) to yield a 1.2 M solution in 4:1 DMF/DMSO. PbI_2_ (581 mg, 99.9%, TCL) was first dissolved in 0.8 mL of DMF (99.8%, Sigma) and 0.2 mL of DMSO (99.8%, Sigma). This solution was then added to 188 mg of FAI (99.999%, Great Cell Solar) and 28 mg of CsI (99.999%, Sigma) sequentially, allowing for complete dissolution between each step. Once the perovskite solution was fully dissolved, 80 μL was statically deposited onto a substrate and evenly spread using the capillary force of the pipet tip. This solution was then spin-coated in two steps at 1000 rpm for 10 s and 6000 rpm for 20 s. During the second step, 250 μL of chlorobenzene (99.5%, Sigma) was dynamically deposited 26 s after the start of the first step. After quenching with the antisolvent, the substrates were annealed at 150 °C for 10 min.

At least 2 h after depositing the perovskite layer, the top passivation layer was deposited in the glovebox. The gap between the depositions was to ensure full removal of the perovskite solvents from the spin coater, which could damage the film surface during the deposition of the top layers. Solutions of 1 mg/mL PEAI (Dyenamo), AMAI, and AEAI ammonium salts in IPA (anhydrous, Sigma) were prepared and allowed to dissolve fully before deposition. For the anthracene compounds, heating at 100 °C was required to aid dissolution; 80 μL was dynamically spin-coated at 5000 rpm for 20 s. Half of the samples for each passivation variation were subsequently annealed at 100 °C for 40 min to investigate the accelerated aging effect of a postdeposition thermal treatment.

For the hole-transport layer, 91 mg of spiro-OMeTAD (1-Material) was first dissolved in 1 mL of chlorobenzene (anhydrous, Sigma), yielding a 0.07 M solution. Two dopant solutions were prepared by dissolving 517 mg of Li-TSFI (Sigma) in 1 mL of acetonitrile (anhydrous, Sigma) and 376 mg of FK 209 Co(III) TFSI (98%, Sigma) in 1 mL of acetonitrile (anhydrous, Sigma). 16.5 μL of the 1.8 M Li-TSFI solution, 36 μL of 4-*tert*-butylpyridine (Sigma-Aldrich, 98%), and 9 μL of the 0.25 M FK 209 Co(III) TFSI solution were added to the 1 mL spiro-OMeTAD solution sequentially, and 80 μL of this solution was dynamically spin-coated at 3000 rpm for 30 s.

The edges of the substrates were then cleaned using cotton swabs to remove the outer 3 mm of material with DMF (99.8%, Sigma) and ACN (99.5%, Sigma). This step enables electrical contact with the bottom FTO layer. The gold anode was then deposited from a gold evaporation slug (99.99% trace metals basis, Sigma) in a thermal evaporation chamber (NANO 36, Kurt J. Lesker Company), resulting in a 50 nm gold layer.

### Device Characterization

The PCE of the devices was measured with a parallel JV system (Litos Lite, Fluxim) in conjunction with a sun simulator (SINUS-70, Wavelabs). All cells had masks with 2.5 mm × 2.5 mm openings to define a pixel area exposed to light of 0.0625 cm^2^ per pixel. Dark and light parallel JV curves were measured from 1.4 to −0.5 V at a scan rate of 50 mV/s. Sequential scans from positive to negative and from negative to positive voltages were acquired. The light parallel JV curves were recorded holding the cell under illumination at AM1.5 conditions, the incident light intensity calibrated with a reference Si solar cell. The stabilized PCE was measured during a 1 min stress test at the MPPT.

To characterize the device performance, key values were extracted from the forward and reverse *J*–*V* curves. The maximum power point current and voltage occur at the values that result in maximum power output. Comparing this maximum power to the theoretical value resulted in the calculation of the fill factor (FF) using eq 1. From here, the power conversion efficiency (PCE) was calculated with eq 2.^33^ The *hysteresis index* is a measure of this variability and was calculated using eq 3. The stabilized PCE was determined by tracking the performance over 60 s at the maximum power point conditions. Averaging the last three points of these measurements gives the stabilized PCE of the device.
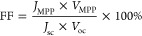
1
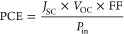
2
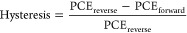
3

For each of the PEAI 0, AMAI 0, AEAI 0, and AMAI 40 device variations, the stability of the best performing solar cells was evaluated with an accelerated stress test in a Litos setup from Fluxim. The solar cells were held at the MPPT during the measurement under constant 1 Sun equivalent illumination and elevated temperature. Devices were tested for 335 h at 65 °C and then for an additional 150 h at 85 °C. Via this measurement, the effect of the passivation treatment on the thermal stability of the devices can be investigated. The stability curves of pixels that maintained nonzero PCE were then averaged and plotted for each device, considering only these working pixels to be viable for analysis.

### Thin-Film Preparation

Before depositing the perovskite film, the substrates were prepared following the same cleaning protocol as that was used for full devices. Unpatterned FTO on glass was first cut into 1 in. × 1 in. substrates and then scrubbed with 10% (v/v) Mucasol (diluted in DI water, Sigma). After rinsing with DI water, the substrates were then sonicated in 2% (v/v) Mucasol (diluted in DI water, Sigma) for 15 min, rinsed with DI water, and sonicated in DI water, acetone (99.5%, Sigma), and IPA (99.5%, Sigma) for 10 min each. The substrates were then left in IPA until being dried with nitrogen gas. Immediately before film deposition, the substrates were treated with UV-ozone for 15 min.

The 1.2 molar Cs_0.09_FA_0.91_PbI_3_ perovskite solution was prepared by mixing 1.05:0.91:0.09 molar ratios of PbI_2_/FAI/CsI in 4:1 DMF/DMSO in a nitrogen glovebox, following the same protocol as for the full devices. 80 μL was statically deposited onto the ITO substrates and spin-coated in two steps at 1000 rpm for 10 s and at 6000 rpm for 20 s. 250 μL of chlorobenzene (99.5%, Sigma) was dynamically deposited 26 s after the start of the first step. The substrates were then annealed at 150 °C for 10 min after quenching with an antisolvent.

The passivation layer was deposited at least 2 h after depositing the perovskite layer. The 1 mg/mL solutions of PEAI (Dyenamo), AMAI, and AEAI in IPA (anhydrous, Sigma) were prepared and allowed to dissolve fully before deposition. 80 μL was then dynamically spin-coated for 20 s at 5000 rpm. Samples with the thermal treatment variation were subsequently annealed at 100 °C for 40 min.

### Thin-Film Characterization

X-ray photoelectron spectroscopy (XPS) data were obtained from a Thermo K-Alpha XPS system using an aluminum K-Alpha 1.486 keV source. Elemental scans for C 1s, N 1s, O 1s, I 3d, Pb 4f, and Cs 3d were measured in addition to valence and XPS survey scans, as these are the elements expected to be present in the films. The data were analyzed in Thermo Avantage, and the signals were compared to reference peaks in order to determine what elemental species are present on the surface of the films. Integrating the area under these peaks allows for the calculation of relative abundance of each elemental species present to better understand the chemical reactions that occur at the passivation interface.

Grazing-incidence wide-angle X-ray scattering (GIWAXS) data were obtained at beamline 11-BM at NSLSII with 20 s integration time at incident angles of α = 0.050°, 0.100°, and 0.500° using a Dectris Pilatus detector. A 13.5 keV X-ray beam was used, with the beam size of 0.2 mm (horizontal) × 0.05 mm (vertical) and 1 mrad divergence. The energy resolution was at 0.7%. Analysis of the GIWAXS data was performed using the SciAnalysis Python package, which was supplied by the beamline. Samples were held in a vacuum during the measurement and cut to ∼∼4 mm × 4 mm to minimize peak broadening at low incident angles due to the spreading of the beam footprint.^24^

Further analysis was done to examine the ratio of 2D to 3D phases present as a function of incident angle and to estimate the vertical crystallite size. The 2D phase is defined by the peak at 0.34 Å^–1^ for AMAI 0, 0.30 Å^–1^ and 0.34 Å^–1^ for AMAI 40, and 0.30 Å^–1^ for AEAI 0 and AEAI 40, while all 3D phases are defined by the peak at 1.0 Å^–1^. For AMAI 40, the total integrated intensity of both low-angle peaks is used to define the 2D phase. Integrated intensities were calculated by fitting the diffraction data to Gaussian curves with a linear background. This same fit was also used to calculate the full width at half-maximum of each peak and estimate the crystallite size using the Scherrer formula (eq 4), where *D* is the spherical crystal diameter and Δ*Q* is the full width at half-maximum.^34^
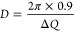
4

The crystallite size is used to estimate the film thickness of the capping layers as they are derived from the radial integration of 1D scattering profiles for −20° < χ < 20° at an incident angle of α = 0.100°. This measurement can also be determined using the ratio of 2D to 3D phases present as a function of incident angle.^35,36^ However, due to the limited number of data points, this model was not used for the present analysis.

### Low-Dimensional Film Preparation

PEAI (62.3 mg) and PbI_2_ (57.6 mg) were dissolved in 500 μL of DMF (anhydrous, Sigma-Aldrich). The solution was left to rest at 90 °C for 1 h. 50 μL of the precursor solutions was then spin-cast onto clean glass substrates at 3000 rpm, 30 s, followed by annealing at 100 °C for 10 min.

13.4 mg of AMAI and 9.2 mg of PbI_2_ were dissolved in 120 μL of DMF (anhydrous, Sigma-Aldrich). The solution was left to rest at 90 °C for 20 min. 45 μL of the precursor solutions was then spin-cast onto clean glass substrates at 3000 rpm, 30 s, followed by annealing at 100 °C for 10 min.

14.0 mg of AEAI and 9.2 mg of PbI_2_ were dissolved in 120 μL of DMF (anhydrous, Sigma-Aldrich). The solution was left to rest at 90 °C for 20 min. 45 μL of the precursor solutions was then spin-cast onto clean glass substrates at 3000 rpm, 30 s, followed by annealing at 100 °C for 10 min.

### Low-Dimensional Film Characterization

X-ray diffraction patterns were collected on a Rigaku SmartLab diffractometer in θ/2θ mode at an incident angle of 0.5°. In all cases, the detector was moved to a 150 mm S–D distance, whereas the source–sample distance is 300 mm. No receiving optics were used, and the detector integrated in 1D mode from a series of horizontal lines 20 mm across. A microarea (MA) selection slit was used to define a smaller beam shape, with the vertical slit size set to 0.2 mm.

### Theoretical Calculations

Spin-polarized density functional theory (DFT) calculations were performed using the Vienna ab initio simulations package (VASP). The electron exchange–correlation was represented by the functional of Perdew, Burke, and Ernzerhof (PBE) of generalized gradient approximation (GGA). The ion–electron interaction was described with the projector augmented wave (PAW) method. A cutoff energy of 400 eV was used for the plane-wave basis set. The energies were converged with a 1 × 10^–4^ eV tolerance, and the forces were optimized to within 0.03 eV/Å. The FAPbI_3_(100) surface with the FAI termination was examined in this work. It was modeled with seven atomic layers with 2 × 2 supercells. A vacuum of 30 Å along the *z*-direction was employed to avoid artificial interactions between images. The three topmost atomic layers of the perovskite slabs, together with the surface-bound ligands, were allowed to relax, while the four bottom atomic layers were kept frozen during the geometry optimizations. The binding energy of each ligand to the surface at one monolayer (1 ML) was defined as Δ*E* = *E*_total_ – (*E*_vacancy_ + *E*_ligand_), where *E*_total_, *E*_vacancy_, and *E*_ligand_ correspond to the total energy of the slab with 1 ML surface-bound ligands, the energy of the ligand-covered slab with one ligand vacancy, and the energy of the isolated ligand, respectively. A more negative value of Δ*E* suggests a stronger binding.

## Supporting Information Available

^1^H NMR spectra for all compounds, supplemental XPS elemental scans, summary of surface compositions, GIWAXS images, GIXRD and XRD patterns, maximum power point tracking, supplemental device performance data, summary of maximum and median performance parameters, *J*–*V* curves, and stability data (PDF)
