# Supplementary material for: Thermally Stable Anthracene-Based 2D/3D Heterostructures for Perovskite Solar Cells
Source: ACS Appl Mater Interfaces. 2024 Dec 27;17(1):1209–20. doi: 10.1021/acsami.4c17382 (PMC11783487; doi:10.1021/acsami.4c17382)
Supplement: Supplementary file 1 — am4c17382_si_001.pdf [file am4c17382_si_001.pdf]

<u>**Supporting Information**</u>

Thermally stable anthracene-based 2D/3D heterostructures for perovskite solar cells

Kathryn Bairley[1], Junxiang Zhang[2], Damara G. Dayton[3], Courtney Brea[4], Pattarawadee Therdkatanyuphong[2,5], Stephen Barlow[2], Guoxiang Hu[1], Michael F. Toney[2,3,6], Seth R. Marder[2,3,6,7], Carlo A. R. Perini[1]*, Juan-Pablo Correa-Baena[1,8]*

[1]School of Materials Science and Engineering, Georgia Institute of Technology, North Ave NW, Atlanta, Georgia 30332, USA

[2]Renewable and Sustainable Energy Institute (RASEI), University of Colorado Boulder, Boulder, Colorado 80309, United States

[3]Materials Science and Engineering Program, University of Colorado Boulder, Boulder, Colorado 80309, United States

[4]Department of Chemistry and Biochemistry, Queens College of the City University of New York, New York, New York 11367, United States

[5]Department of Materials Science and Engineering, School of Molecular Science and Engineering, Vidyasirimedhi Institute of Science and Technology, Wangchan, Rayong 21210, Thailand

[6]Department of Chemical and Biological Engineering, University of Colorado Boulder, Boulder, Colorado 80309, United States

[7]Department of Chemistry, University of Colorado Boulder, Boulder, Colorado 80309, United States

[8]School of Chemistry and Biochemistry, Georgia Institute of Technology, North Ave NW, Atlanta, Georgia 30332, USA

Corresponding author: CARP carperini@gatech.edu; JPCB jpcorrea@gatech.edu

Experimental Details

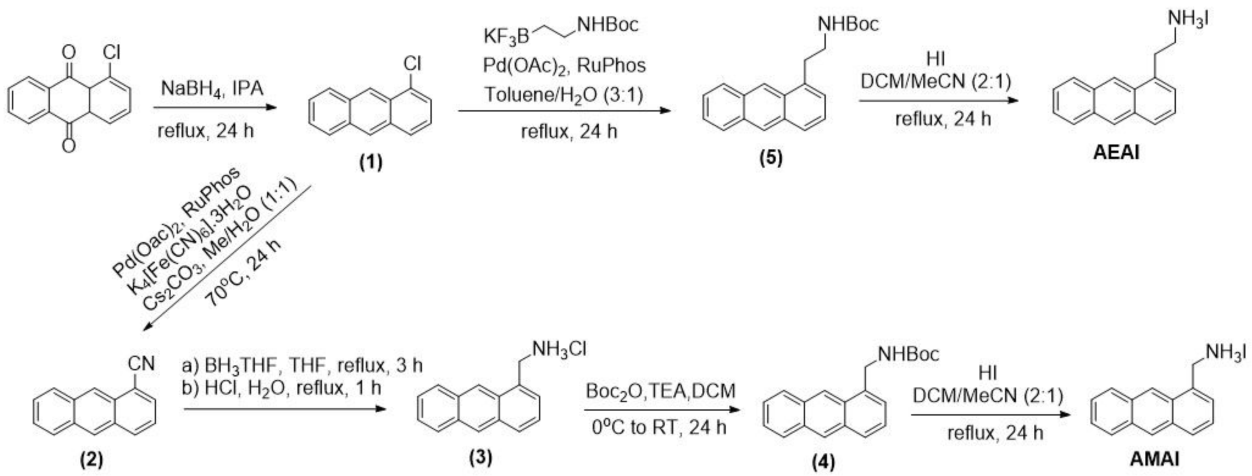

Figure S1. Synthetic procedure of anthracene ammonium iodide derivatives.

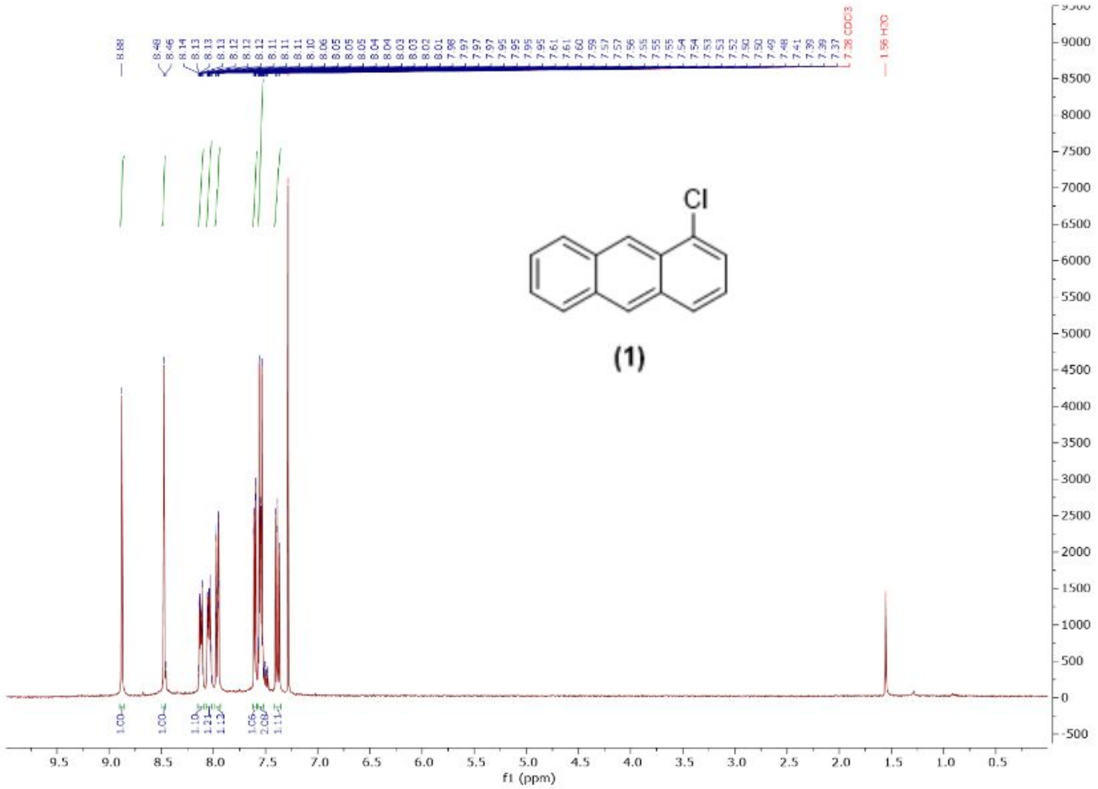

Figure S2. ^{1}H-NMR spectrum of 1-chloroanthracene (**1**) in $CDCl_3$.

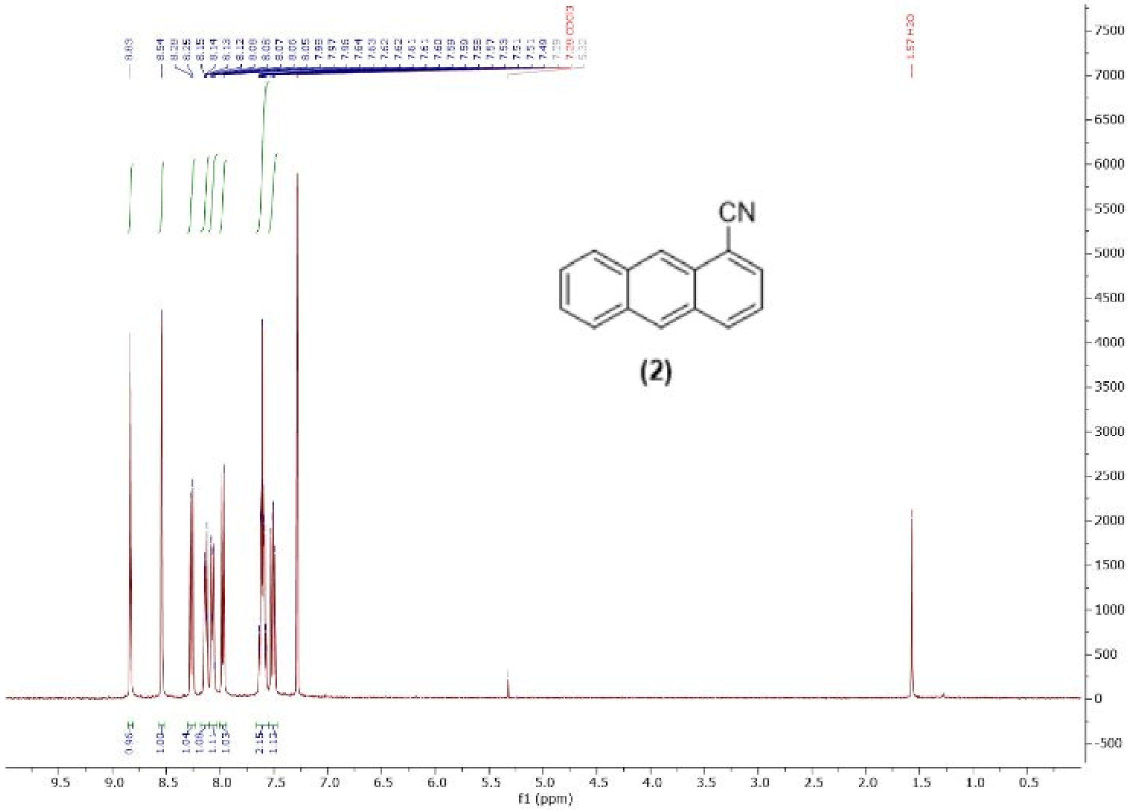

Figure S3. ¹H-NMR spectrum of anthracene-1-carbonitrile (**2**) in $CDCl_3$.

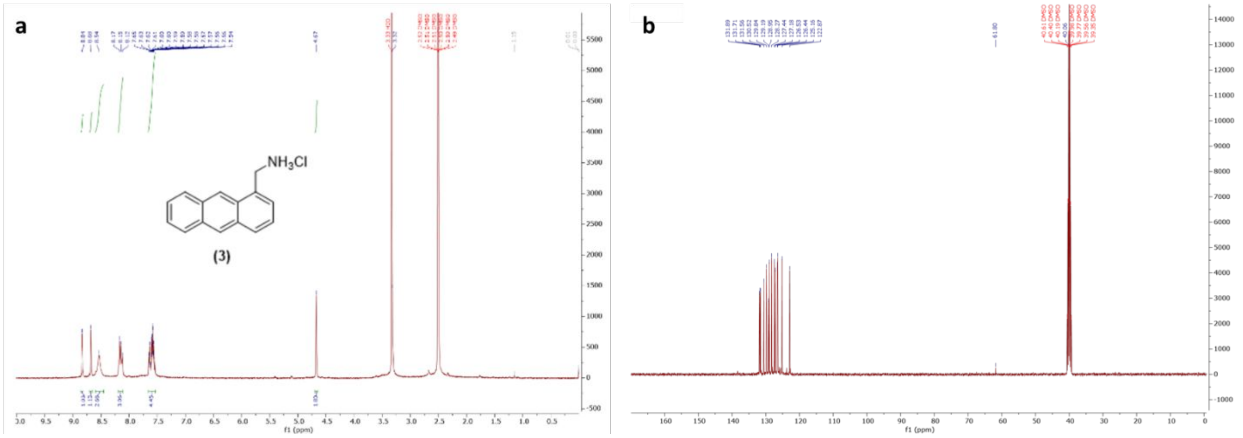

Figure S4. a) ¹H and b) $^{13}C\{^1H\}$ NMR spectra of anthracene-1-ylmethylammonium chloride (**3**) in DMSO-d_6.

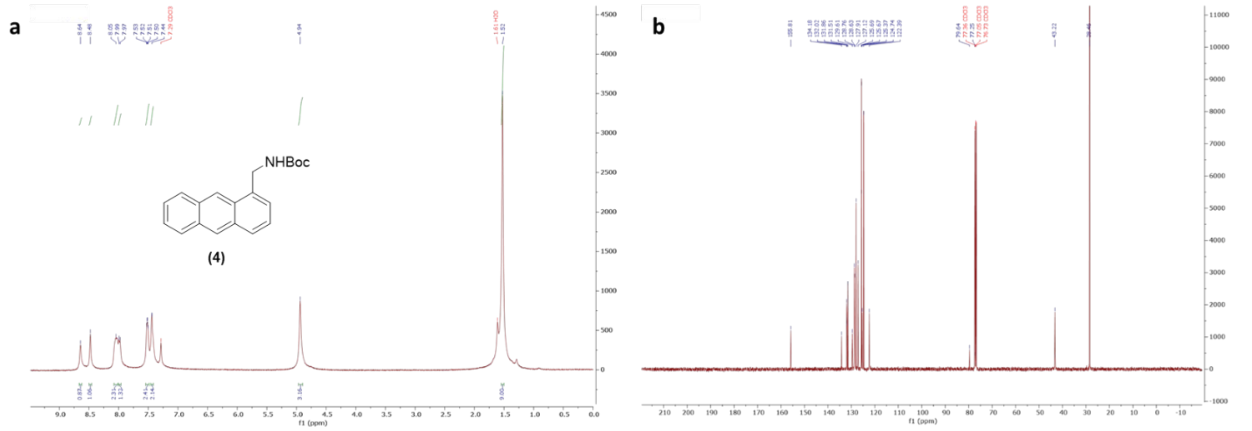

Figure S5. a) ^{1}H and b) $^{13}C\{^{1}H\}$ NMR spectra of *tert*-butyl (anthracen-1-ylmethyl)carbamate (**4**) in $CDCl_3$.

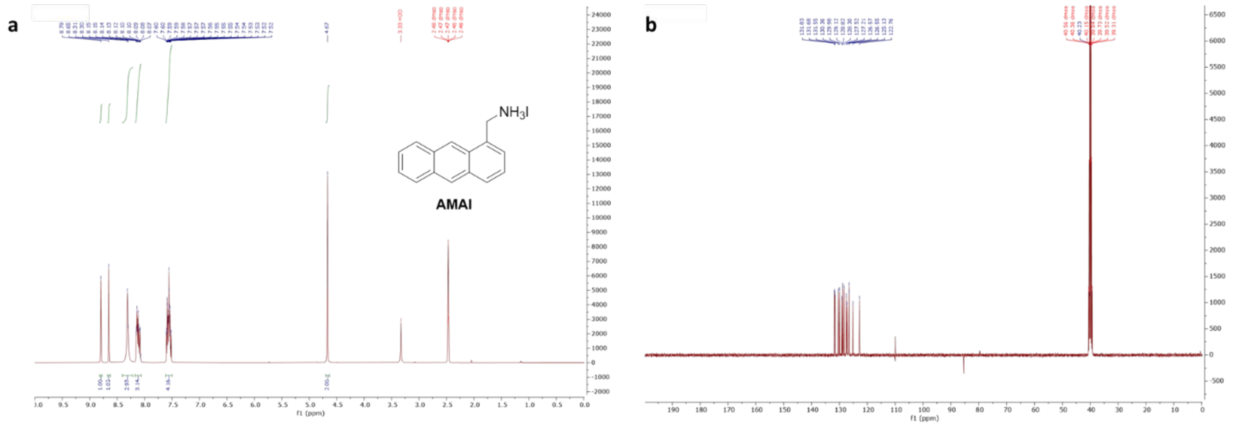

Figure S6. a) ^{1}H and b) $^{13}C\{^{1}H\}$ NMR spectra of AMAI in DMSO-d_6.

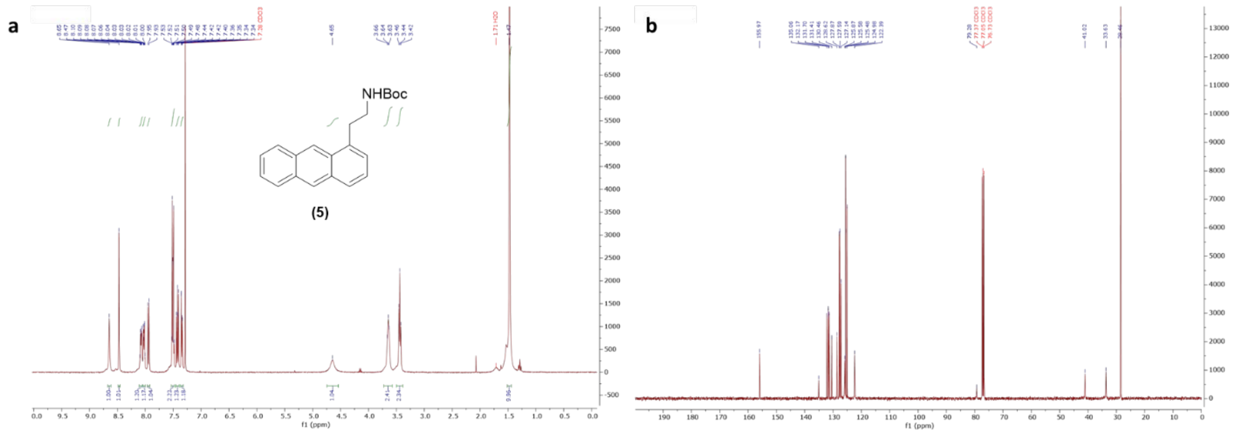

Figure S7. a) ^{1}H and b) $^{13}C\{^{1}H\}$ NMR spectra of *tert*-butyl (2-(anthracen-1-yl)ethyl)carbamate (**5**).

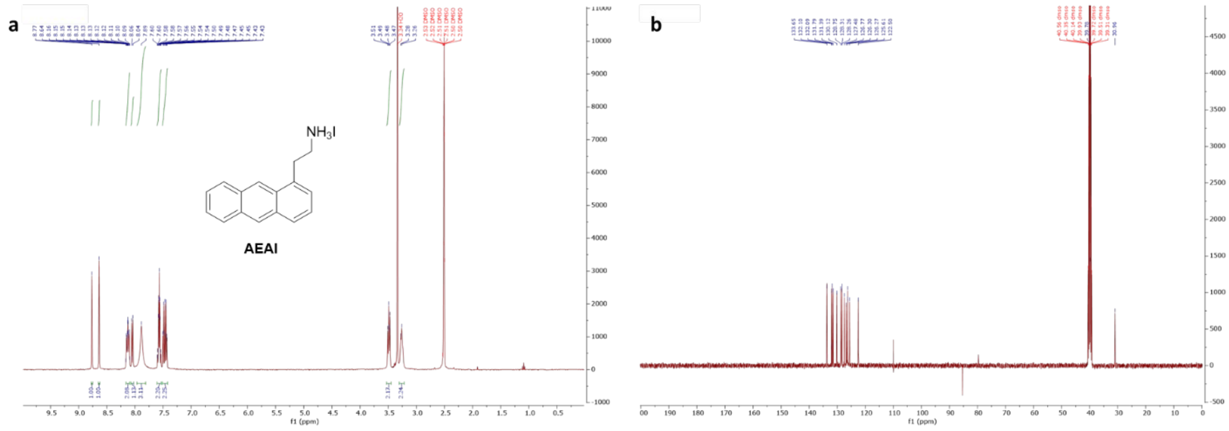

Figure S8. a) ^{1}H and b) $^{13}C\{^{1}H\}$ NMR spectra of AEAI in DMSO-d_6.

Supplemental Data

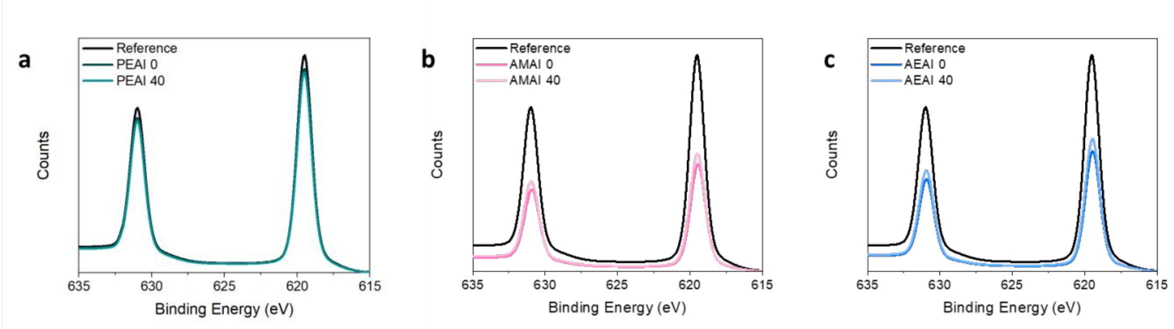

Figure S9. XPS elemental scans of I 3d for (a) PEAI-, (b) AMAI-, and (c) AEAI-treated perovskite films.

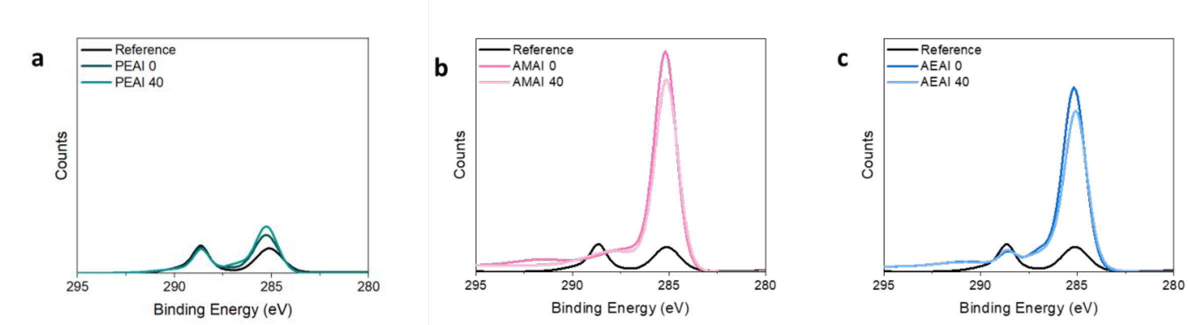

Figure S10. XPS elemental scans of C 1s for (a) PEAI-, (b) AMAI-, and (c) AEAI-treated perovskite films.

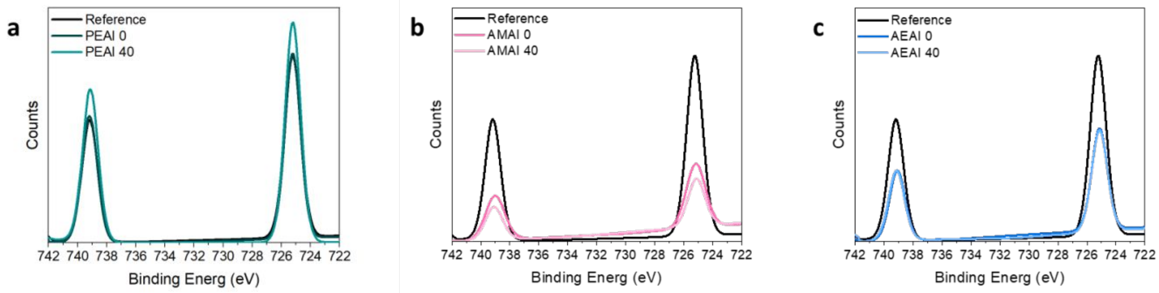

Figure S11. XPS elemental scans of Cs 3d for (a) PEAI-, (b) AMAI-, and (c) AEAI-treated perovskite films. The number after the compound abbreviation refers to the time in minutes the sample was heated at 100 °C.

Table S1. Summary of approximate surface compositions based on integrated XPS peaks. The nitrogen peak at ~402.5 eV is associated with the single bond to carbon in the bulky cations (R-NH_3^+). A second nitrogen peak occurs at ~400.8 eV and correlates to nitrogen bonds in FA^+.

**Composition**	**N (R-NH_3^+)**	**N (FA^+)**	**Cs**	**Pb**	**I**
**Ref [%]**	0.00	26.92	1.13	13.85	58.11
**[X/Pb]**	0.00	0.97	0.08	1.00	4.20
**PEAI 0 [%]**	0.00	26.33	1.19	13.29	58.43
**[X/Pb]**	0.00	0.99	0.09	1.00	4.40
**PEAI 40 [%]**	0.00	25.55	1.46	13.07	58.32
**[X/Pb]**	0.00	0.98	0.11	1.00	4.46
**AMAI 0 [%]**	8.49	22.67	0.91	13.44	54.48
**[X/Pb]**	0.63	0.84	0.07	1.00	4.05
**AMAI 40 [%]**	5.46	22.58	0.57	15.20	56.19
**[X/Pb]**	0.36	0.74	0.04	1.00	3.70
**AEAI 0 [%]**	6.90	23.83	1.04	13.56	54.67
**[X/Pb]**	0.51	0.88	0.08	1.00	4.03
**AEAI 40 [%]**	5.79	24.21	0.99	13.42	55.59
**[X/Pb]**	0.43	0.90	0.07	1.00	4.14

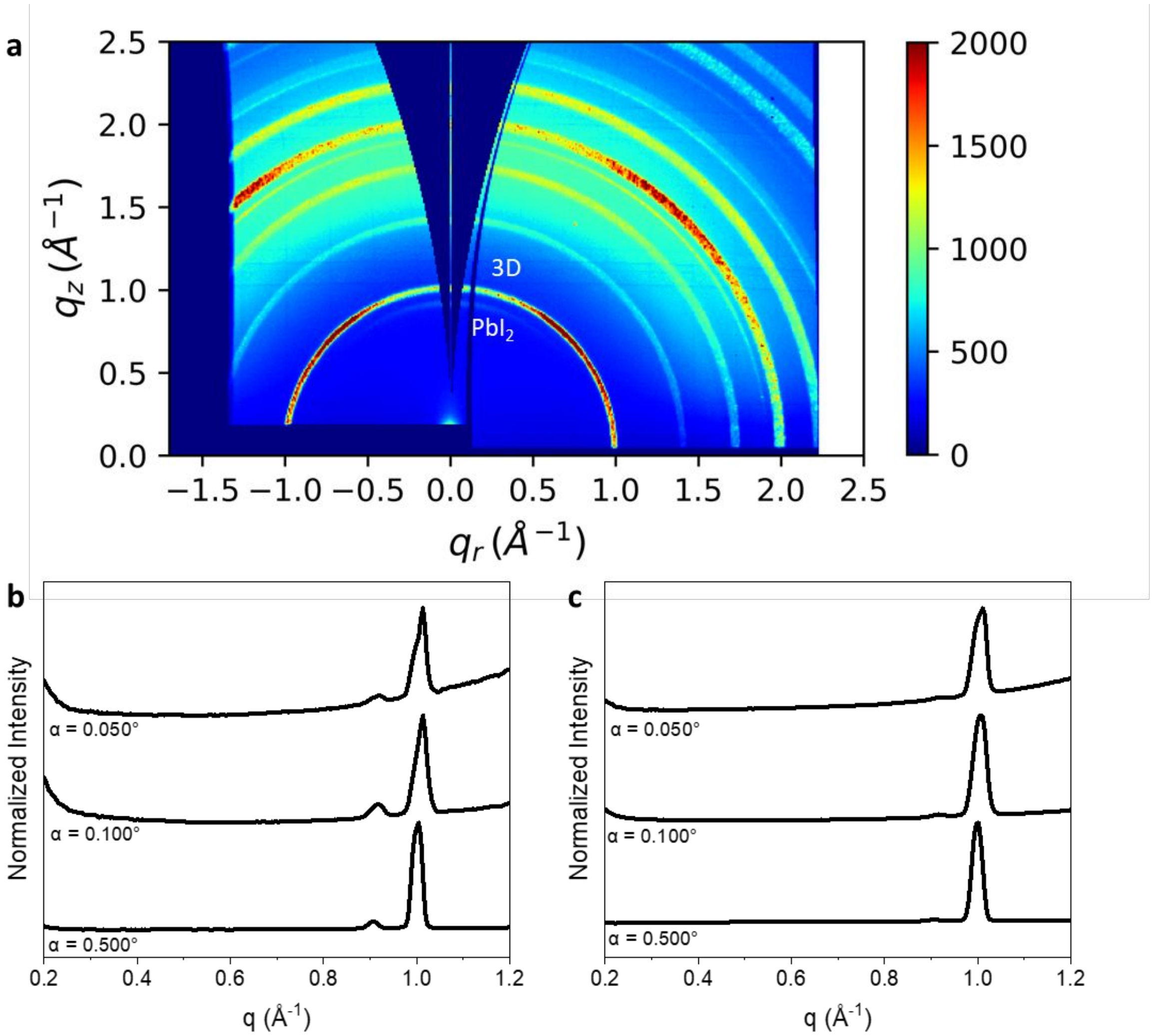

Figure S12. a) GIWAXS patterns of the pristine $Cs_{0.09}FA_{0.91}PbI_3$ film on a FTO substrate at α = 0.100°. b) Radial integration of 1D scattering profiles from the 2D GIWAXS for -20° < χ < 20° and c) -90° < χ < 90°as a function of incidence angle.

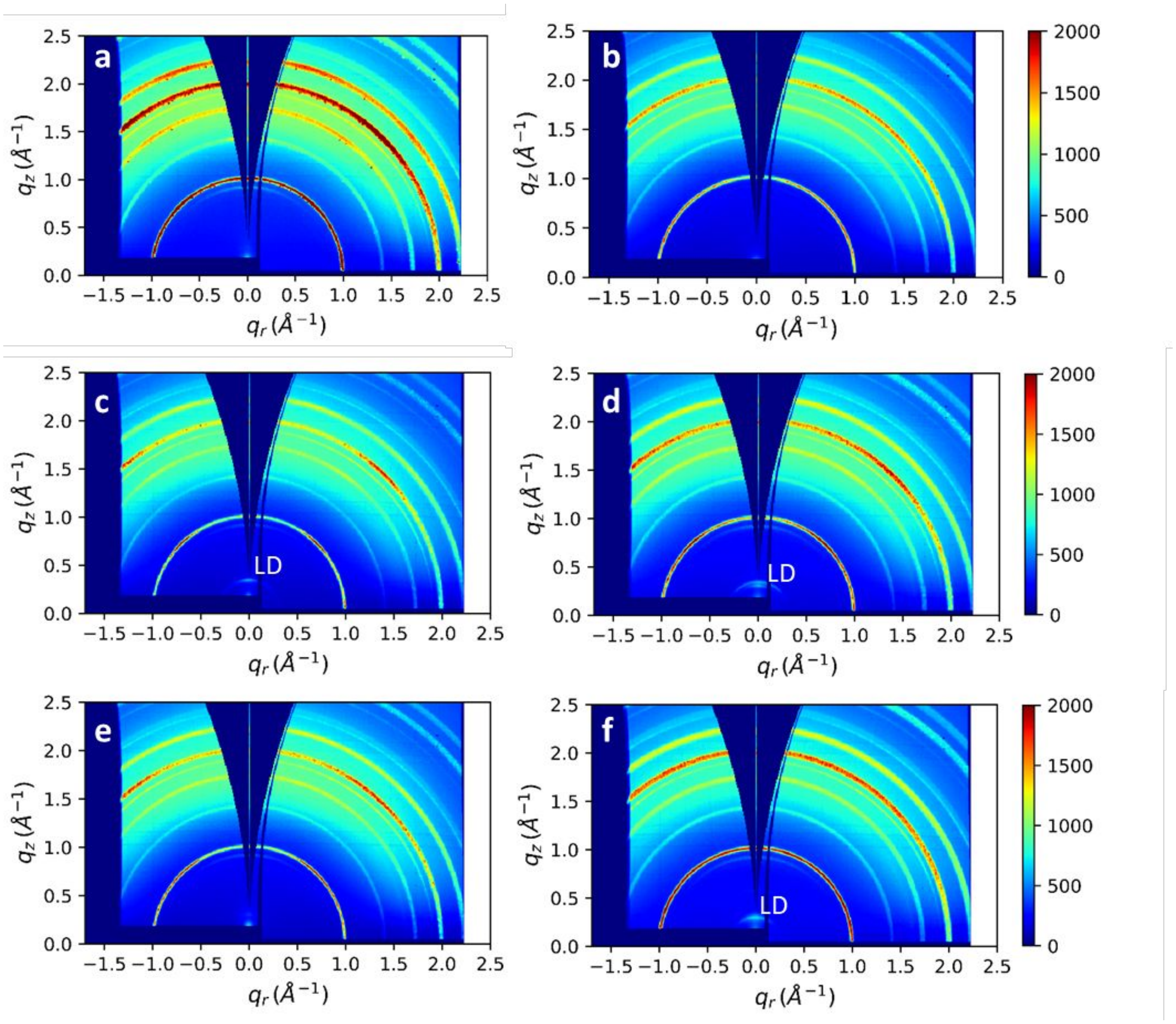

Figure S13. 2D GIWAXS plots for a) PEAI 0, b) PEAI 40, c) AMAI 0, d) AMAI 40, e) AEAI 0, and f) AEAI 40 capping layers on $Cs_{0.09}FA_{0.91}PbI_3$ perovskite films on a FTO substrate at an incidence angle of α = 0.100°. 'LD' stands for low dimensional structures.

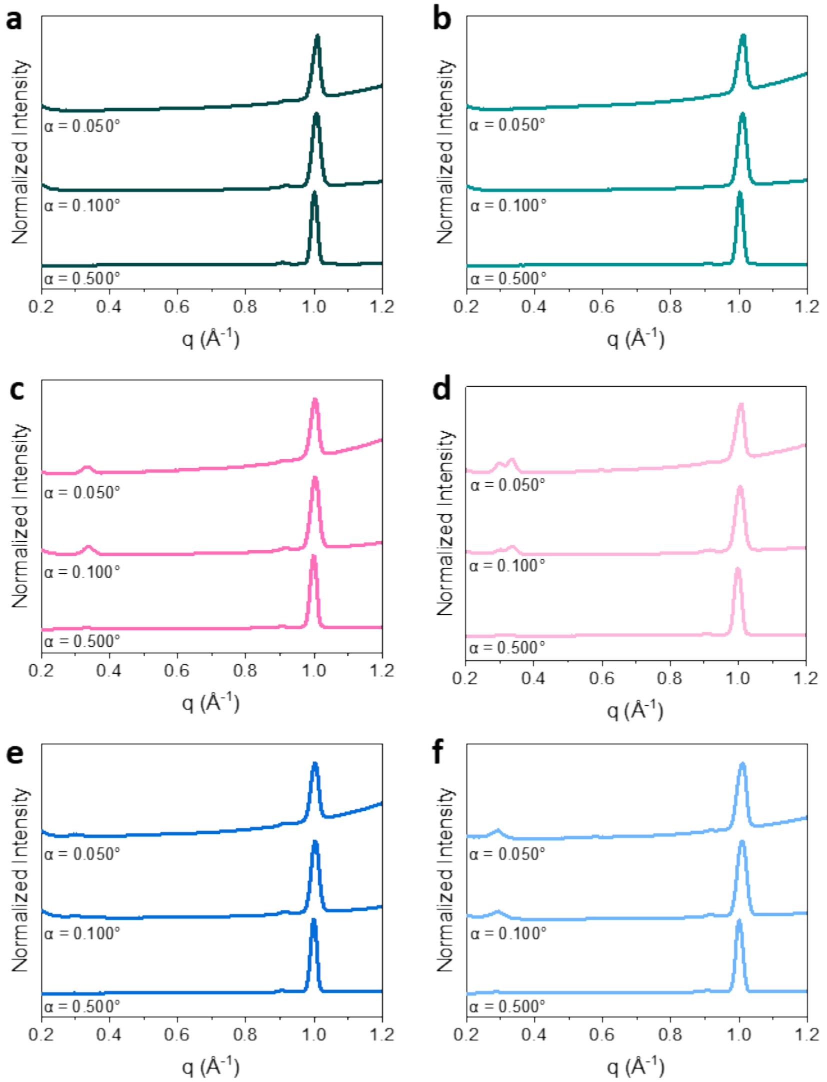

Figure S14. Radial integration of 1D scattering profiles from the 2D GIWAXS for $-90° < \chi < 90°$ as a function of incidence angle for a) PEAI 0, b) PEAI 40, c) AMAI 0, d) AMAI 40, e) AEAI 0, and f) AEAI 40. All data is normalized with respect to the 3D perovskite peak at q=1.0 $Å^{-1}$ and is offset for clarity.

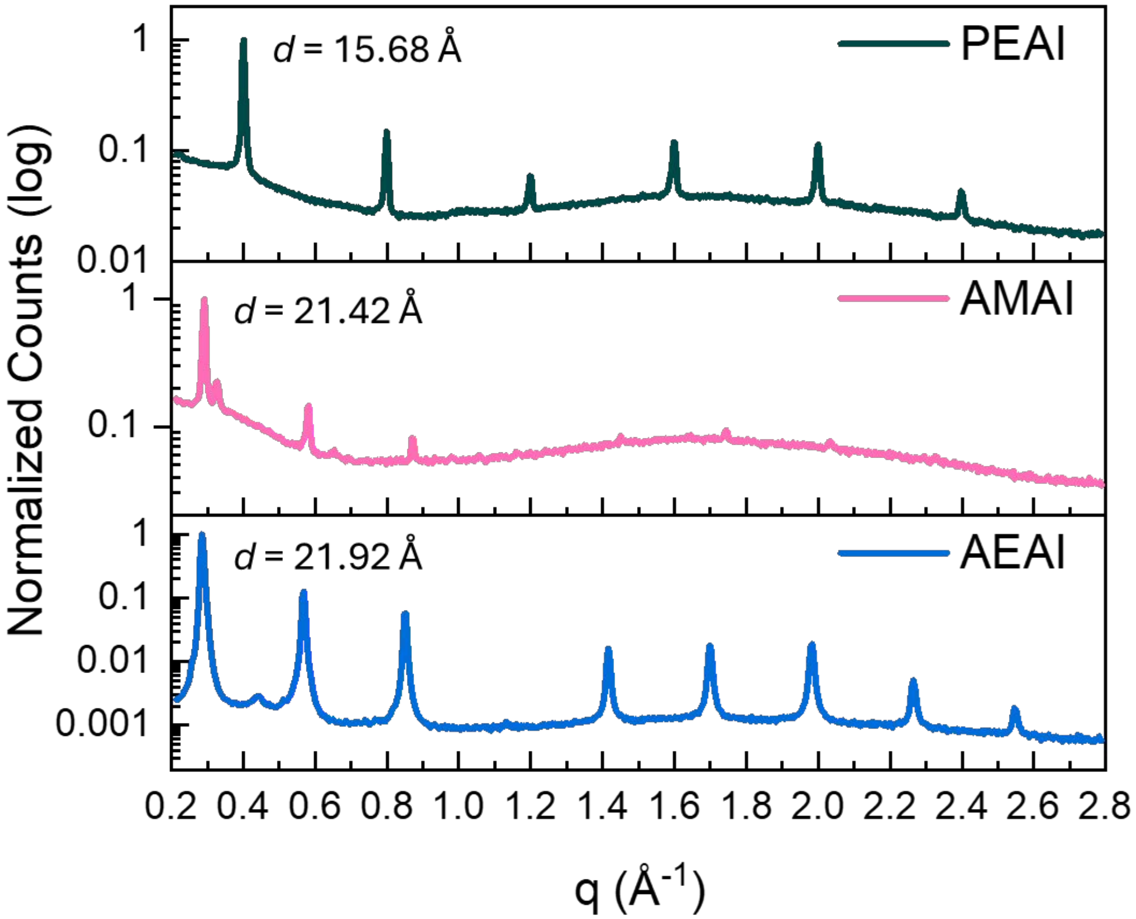

Figure S15. X-ray diffraction peaks of 2D perovskite films and calculated interplanar d spacing as retrieved from the first diffraction peak of each film.

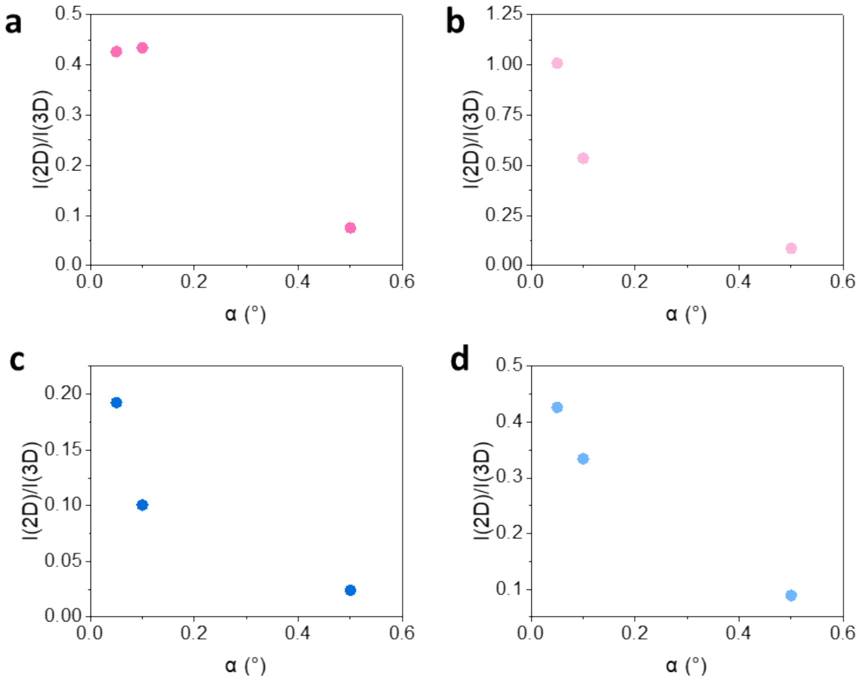

Figure S16. Ratio of integrated intensity of 2D and 3D perovskite peaks of the radial integration of 1D scattering profiles for -20° < χ < 20° as a function of incidence angle for a) AMAI 0 and b) AMAI 40, c) AEAI 0, and d) AEAI 40 films. Sample data from PEAI 0 and PEAI 40 are omitted because the diffraction peak corresponding to the 2D phase is below the limit of detection for all films except PEAI 0 at an incidence angle of α = 0.500°.

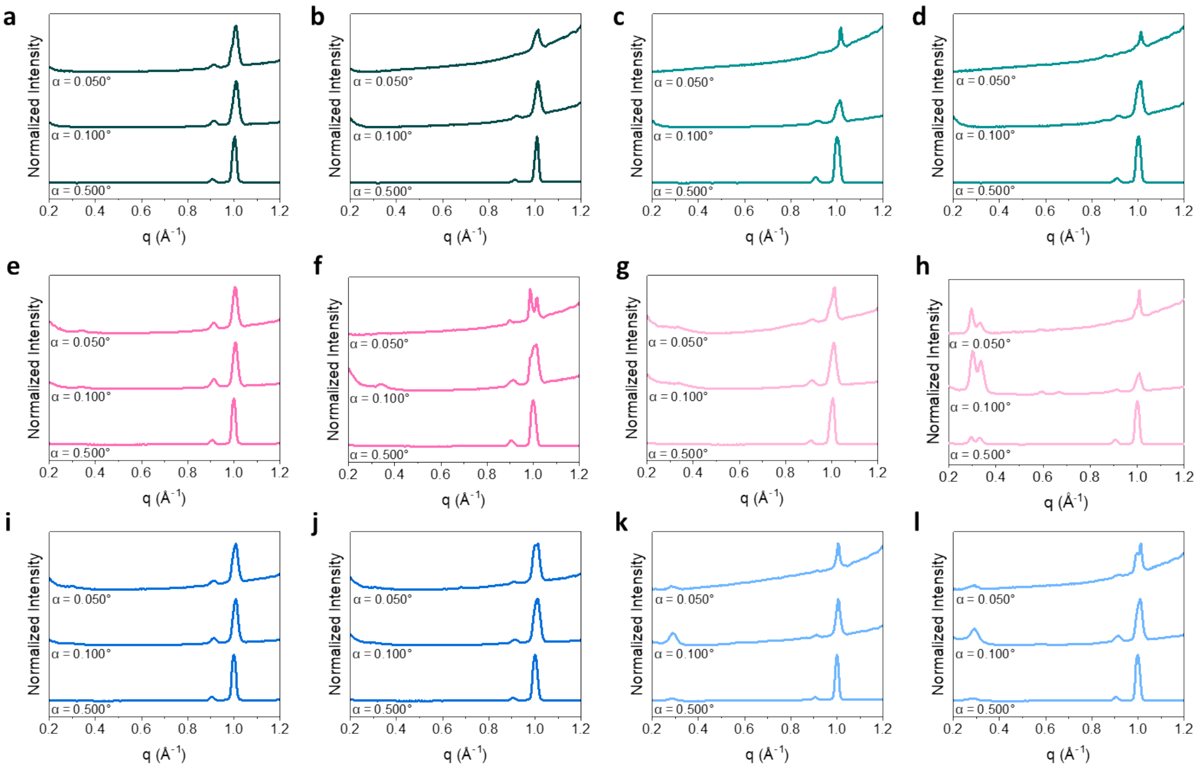

Figure S17. Sample to sample variation of the radial integration of 1D scattering profiles for -20° < χ < 20° as a function of incidence angle for a-b) PEAI 0, c-d) PEAI 40, e-f) AMAI 0, g-h) AMAI 40, i-j) AEAI 0, and k-l) AEAI 40 films. All data are normalized with respect to the 3D perovskite peak maximum intensity at q = 1.0 Å^{-1} and are offset for clarity.

Table S2. Summary of the vertical crystallite sizes of the 2D capping layers calculated from the full width at half maximums of the radial integration of 1D scattering profiles for -20° < χ < 20° at an incidence angle of α = 0.100°. Sample data from PEAI 0 and PEAI 40 are omitted as there is no visible peak in either profile.

Sample	Peak Position [Å^{-1}]	FWHM [Å^{-1}]	Crystallite Size [Å]
**AMAI 0**	0.34	0.032	177
**AMAI 40**	0.30	0.028	199
	0.34	0.034	166
**AEAI 0**	0.30	0.033	171
**AEAI 40**	0.29	0.044	128

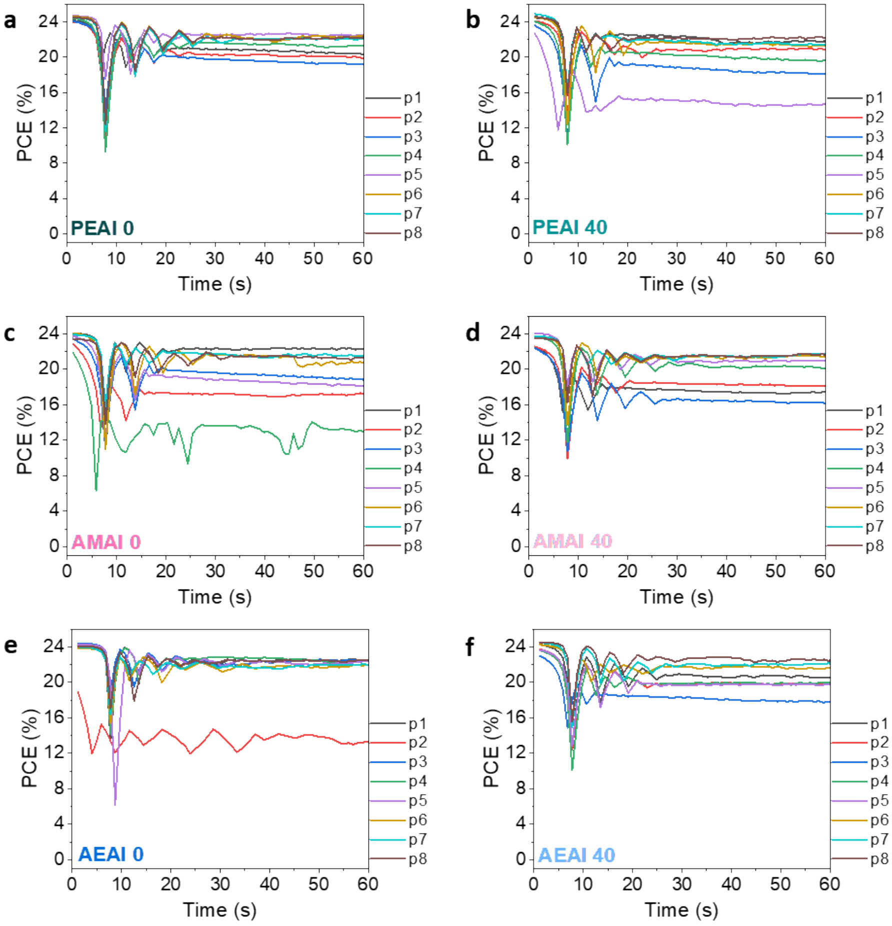

Figure S18. Maximum power point tracking for a) PEAI 0, b) PEAI 40, c) AMAI 0, d) AMAI 40, e) AEAI 0, and f) AEAI 40. These *PCE* measurements were taken over a 60 s interval at the maximum power point to determine the *stabilized PCE*. The plots contain data for one device of each capping variation, with each device containing 8 pixels.

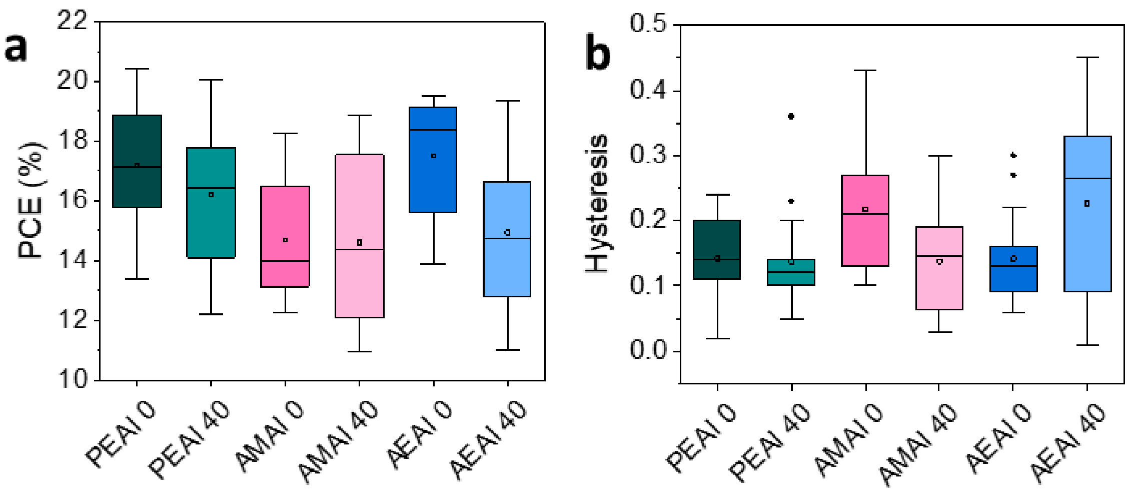

Figure S19. Box plots of (a) power conversion efficiency and (b) hysteresis for each passivation variation calculated from the reverse JV curves. The 0 and 40 that follow the name of the bulky cation refer to the time in minutes for which the films were annealed at 100 °C.

Table S3. Summary of maximum (bolded) and median (± median of absolute deviation) performance parameters. Values are based on reverse *J-V* scans.

Variation	V_{OC} [V]	J_{SC} [mA/cm^2]	FF [%]	PCE [%]	Stabilized PCE [%]	Hysteresis
**PEAI 0**	**1.06**<br>1.00 ± 0.02	**25.1**<br>24.6 ± 0.4	**78.7**<br>69.8 ± 4.3	**20.5**<br>17.1 ± 1.8	**19.5**<br>16.6 ± 1.4	**0.24**<br>0.14 ± 0.6
**PEAI 40**	**1.08**<br>1.00 ± 0.02	**24.9**<br>24.3 ± 0.3	**77.7**<br>66.1 ± 5.5	**20.0**<br>16.4 ± 5.5	**19.0**<br>15.8 ± 2.1	**0.36**<br>0.12 ± 0.02
**AMAI 0**	**1.03**<br>0.96 ± 0.02	**24.6**<br>23.7 ± 0.4	**76.4**<br>61.5 ± 4.4	**18.3**<br>14.0 ± 1.4	**16.8**<br>12.9 ± 2.1	**0.43**<br>0.21 ± 0.07
**AMAI 40**	**1.06**<br>1.00 ± 0.04	**24.7**<br>23.3 ± 0.5	**75.0**<br>62.1 ± 8.5	**18.9**<br>14.4 ± 2.8	**17.7**<br>14.7 ± 2.3	**0.30**<br>0.15 ± 0.06
**AEAI 0**	**1.08**<br>1.02 ± 0.02	**24.6**<br>24.3 ± 0.2	**76.9**<br>73.3 ± 2.8	**19.5**<br>18.4 ± 1.0	**18.9**<br>18.1 ± 0.3	**0.30**<br>0.13 ± 0.4
**AEAI 40**	**1.05**<br>0.98 ± 0.03	**24.6**<br>23.9 ± 0.4	**75.0**<br>64.8 ± 5.4	**19.3**<br>14.7 ± 1.9	**18.5**<br>14.3 ± 1.4	**0.45**<br>0.27 ± 0.09

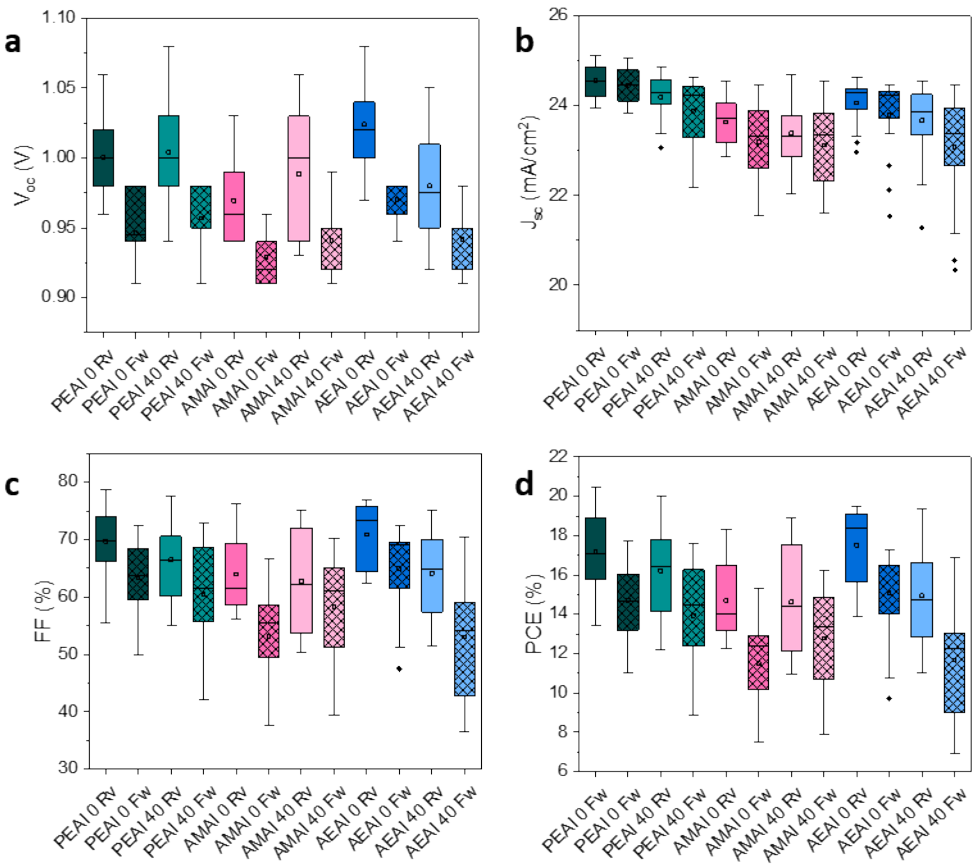

Figure S20. Box plots of (a) open-circuit voltage, (b) short-circuit current, (c) fill factor, and (d) power conversion efficiency for each capping variation. The data presented in solid boxes are based on reverse *J-V* scans, while the hashed boxes are based on forward *J-V* scans.

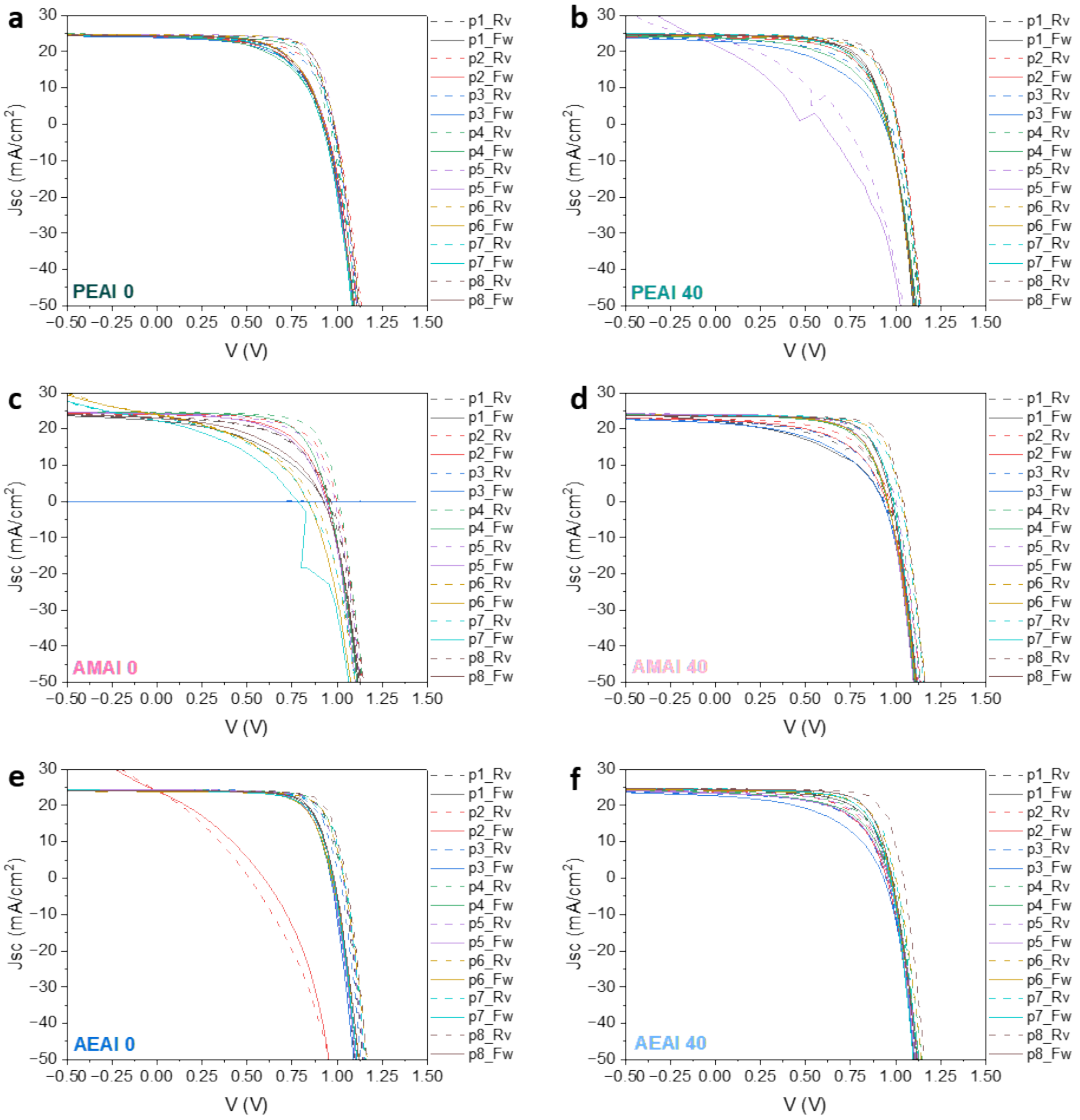

Figure S21. *J-V* curves for a) PEAI 0, b) PEAI 40, c) AMAI 0, d) AMAI 40, e) AEAI 0, and f) AEAI 40. The plots contain data for one device of each capping variation, with each device containing 8 pixels. The dashed lines contain data from the reverse *J-V* scans, while the solid lines are based on forward *J-V* scans.

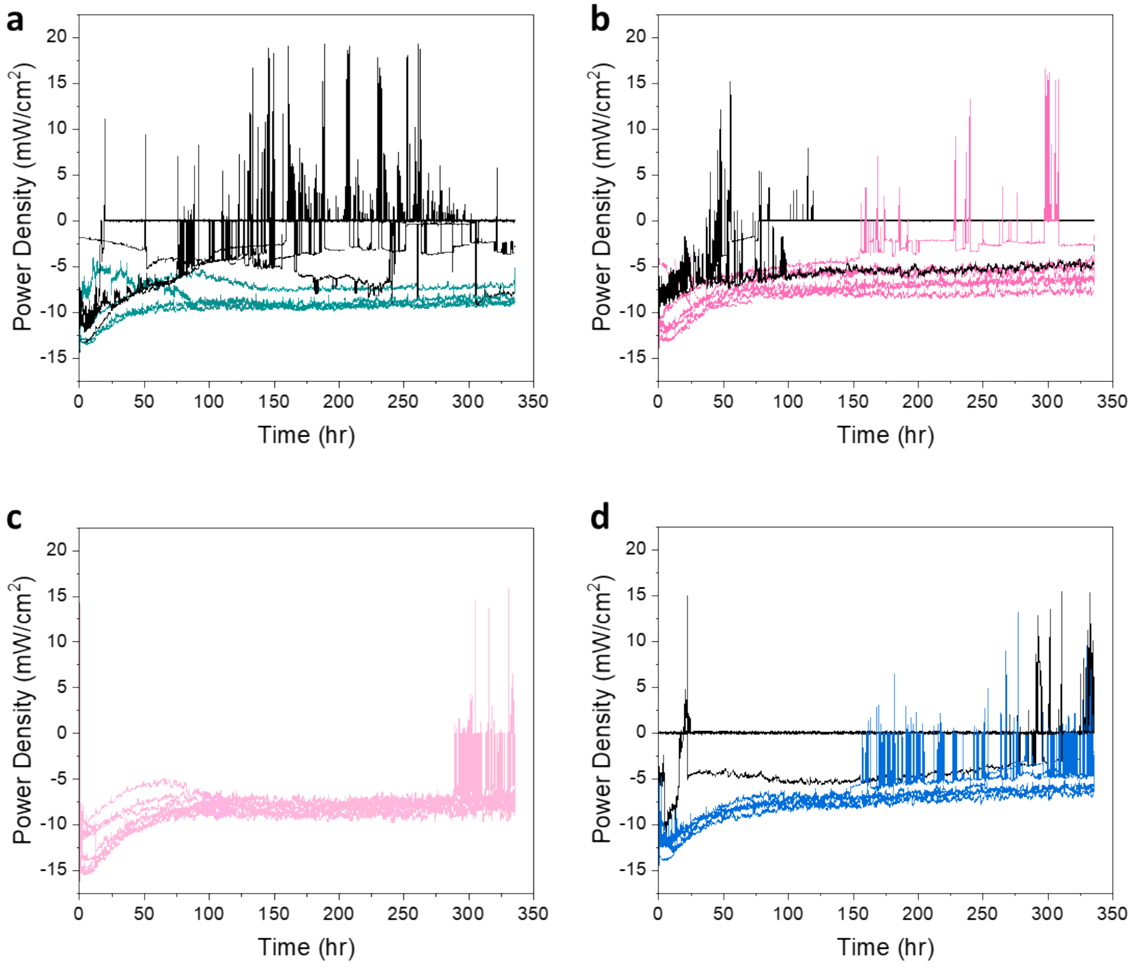

Figure S22. Thermal stability data of pixels at 65°C with a) PEAI 0, b) AMAI 0, c) AMAI 40, and d) AEAI 0 passivation layers. Pixels plotted in black are considered non-working, while pixels plotted in the corresponding treatment color are "viable".
